# Plectin promotes an aggressive phenotype and represses cytotoxic T cell activity in pancreatic cancer

**DOI:** 10.3389/fimmu.2026.1894080

**Published:** 2026-08-17

**Authors:** Cody L. Wolf, Roxanne K. Ruiz, Sokchea Khou, Robert Cornelison, Edward B. Stelow, Karl M. Kowalewski, Matthew J. Lazzara, Amanda Poissonnier, Lisa M. Coussens, Kimberly A. Kelly

**Affiliations:** 1 Department of Biomedical Engineering, University of Virginia, Charlottesville, VA, United States; 2 Department of Molecular Physiology and Biological Physics, University of Virginia, Charlottesville, VA, United States; 3 Department of Cell, Developmental, & Cancer Biology, Oregon Health and Science University, Portland, OR, United States; 4 Department of Experimental Pathology, University of Virginia, Charlottesville, VA, United States; 5 Department of Chemical Engineering, University of Virginia, Charlottesville, VA, United States; 6 Knight Cancer Institute, Oregon Health and Science University, Portland, OR, United States

**Keywords:** cancer immunology, pancreatic cancer, plectin, therapeutic target, tumor microenvironment

## Abstract

**Background:**

Pancreatic adenocarcinoma (PDAC) is an abysmal disease with poor clinical outcomes, largely due to limited life-extending treatments. Notably, PDAC displays a T cell-suppressive tumor microenvironment, and the underlying molecular mechanisms that lead to this phenotype remain poorly understood.

**Methods:**

Utilizing the TCGA-PAAD dataset, tumor samples were separated by PLEC expression to evaluate patient survival, and pathway analyses associated with increased tumorigenesis. Evaluation of immune infiltration and subsequent immune deconvolution was performed using tidyestimate and CIBERSORTx R packages and immunohistochemistry (IHC) from human PDAC samples was performed to analyze PLEC expression and immune cell infiltration. Single-cell RNA-seq (scRNA-seq) analysis from 229 PDAC patients was analyzed to investigate signaling dynamics and immune cell infiltration in PLEC^High^ patients. Functional validation was provided using a monoclonal antibody (mAb) against cell surface plectin (CSP) in two murine PDAC models to examine changes in tumor growth and immune cell subset abundance.

**Results:**

Our studies revealed that high plectin expression results in an overall worse survival associated with activation of pro-tumorigenic pathways and decreased anti-tumor immune signature in PDAC patients. Analysis via GSEA indicates PLEC^High^ patients display an aggressive phenotype and suppressed pro-inflammatory signaling pathways. Immune ESTIMATE scores were significantly decreased in PLEC^High^ patients, and IHC and scRNA-seq analysis revealed that PLEC^High^ tumors display a decrease in anti-tumor CD8^+^ T cells. *In vivo* analyses using an anti-CSP mAb revealed a reduction in tumor growth kinetics compared to IgG control corresponding with a significant increase in proliferating and activated cytotoxic CD8^+^ T cells. Anti-CSP-mediated tumor suppression was inhibited when CD8^+^ T cells were depleted, indicating that anti-CSP treatment is contingent on cytotoxic T cell functionality.

**Discussion:**

Our findings identify plectin as a biomarker of aggressive disease in PDAC, with high plectin expression associated with decreased T cell infiltration, and anti-CSP treatment reinstates antitumor immunity and decreases tumor volume *in vivo*. These findings suggest that plectin is a novel therapeutic target with the potential to enhance immune responses in PDAC and improve patient outcomes.

## 1 Introduction

Pancreatic adenocarcinoma (PDAC) is a devastating disease with a median survival of 6 months and a 5-year relative survival rate of only 13% (1). In 2026, 67, 530 new PDAC cases and 52, 740 deaths are estimated in the United States, and it is projected to be the 2^nd^ leading cause of cancer-related deaths by 2030 (1, 2). Owing to late-stage diagnosis and limited therapeutic options for patients, treatment relies primarily on surgery and chemotherapy (3, 4). PDAC has been historically categorized as an “immunologically cold” tumor, making it challenging to treat with immune checkpoint blockade therapy, which has revolutionized cancer treatment across many subtypes (5, 6). Understanding the molecular mechanism behind the “immunologically cold” phenotype of PDAC and identifying novel targets that are clinically actionable for most patients is crucial to improving therapeutic outcomes.

Previously, we identified plectin as a prognostic biomarker for PDAC (7). Plectin is a 500 kDa cytolinker protein within the plakin family encoded by the gene *PLEC* and is known to interact with all three components of the cytoskeleton to stabilize intracellular components, adhesion complexes, and organelles (8, 9). In recent years, we and others have reported that plectin is highly expressed in numerous malignancies (7, 10–16), including both primary and metastatic cancers (15) and is a significant indicator of worse overall survival (17–19). Loss-of-function studies have demonstrated that plectin positively regulates classical hallmarks of cancer, including proliferation, migration, adhesion, invasion, and tumor formation (10, 20, 21). Importantly, our group previously reported that plectin is aberrantly mislocalized to the plasma membrane in aggressive cancers (7, 15, 20) and demonstrated that pharmacological inhibition of membrane-localized plectin (cell surface plectin (CSP)) reduced tumor cell proliferation and migration through MAPK signaling (16). Critically, CSP expression is high and ubiquitous in 92% of PDAC tumor samples, while remaining absent in normal tissue (7, 15, 22), implying CSP is a viable target for therapeutic intervention for CSP-positive tumors. Based on these findings, we developed a first-in-class CSP-targeting monoclonal antibody that induces cytotoxicity and decreases cell migration in CSP+ ovarian cancer (14, 16). Most recently, a Phase I/II dose escalation, all-comers clinical trial evaluating a human anti-CSP mAb with patients presenting with advanced-stage cancer of a variety of malignancies showed that targeting CSP is safe, and six patients reached stable disease ranging from 11–33 weeks (3, PDAC, 2 ovarian cancer, and 1 cholangiocarcinoma) (23).

While research on the role of plectin in cancer has grown exponentially over the past two decades, the specific mechanism by which plectin promotes cancer progression and decreases survival remains to be fully elucidated. To further examine the role of plectin in PDAC, we performed differential gene expression and pathway analyses using the TCGA-PAAD dataset to identify unique tumorigenic pathways in PLEC^High^ tumors. Here, we uncovered that in addition to its roles in proliferation and migration, plectin expression correlates with an “immunologically cold” phenotype in PDAC with suppressed anti-tumoral immune signaling pathways. High plectin expression, as assessed via bulk and scRNA-seq, was associated with reduced anti-tumor CD8^+^ T cell infiltration, which was reversed upon pharmacological inhibition of CSP, indicating that plectin mislocalization to the membrane contributes to the immune-cold phenotype in PDAC. Together, these studies have identified a novel role for plectin in the tumor microenvironment and represent a new target to unlock the potential of immunotherapies in PDAC.

## 2 Materials and methods

### 2.1 Bulk RNA-seq differential gene expression and gene set enrichment analysis

RNA-seq transcriptomic data and clinicopathological features of 185 PAAD patients were acquired from the Genomic Database Commons (https://portal.gdc.cancer.gov/) using the Bioconductor package TCGAbiolinks. Samples were stratified into quartiles based on normalized *PLEC* counts. Patient survival between low and high groups were analyzed using the survminer package (v.0.5.1). Statistical significance was calculated using long-rank test. Differential gene expression analysis was performed using the DESeq2 package (v1.50.2) (24). Before running DESeq2, the gene matrix was pre-filtered by removing low counts, and log2fold change shrinkage and variance-stabilizing transformations were employed (25–27). Gene set enrichment analysis (GSEA) was performed using the clusterProfiler (v4.18.4) package (28). The following gene set collections were retrieved from the Molecular Signatures Database (MSigDB) using the msigdbr package (v25.1.1) and used for pathway analysis: Hallmark, GO Biological Process, Reactome, KEGG, WikiPathways, and ImmuneSigDB. The following parameters were applied: minimum and maximum gene set sizes of 10 and 1500, respectively; p-value cutoff of 0.05, with p-values adjusted using the Benjamini-Hochberg (BH) procedure. A fixed random seed was set up for each collection to ensure reproducibility. Furthermore, we utilized the pathfindR package (v2.7.0), an active-subnetwork-oriented pathway enrichment analysis. To reduce the redundancy of enriched terms, we implemented hierarchical clustering, which uses a pairwise distance matrix based on the kappa statistics between the enriched terms [as proposed by Huang et al. (29)], and visualized the results as a bubble plot using the ‘cluster_enriched_terms’ and ‘term_gene_graph’ functions.

### 2.2 Immune cell analysis and deconvolution

Tumor Purity, Immune, Stromal, and ESTIMATE scores were examined using the tidyestimate package (v.1.1.1). For immune deconvolution of bulk RNA-seq samples, sample–gene matrices from each quartile were run on the CIBERSORTx webserver (30) as mixture files. Relative scores were obtained using the following parameters: LM22 signature gene file, 1000 permutations, and disabled quantile normalization. The results were downloaded using default settings. Bar plots of the relative immune fractions and absolute scores were generated using the ggplot2 package (v4.0.2). Wilcoxon tests were performed to determine the significance of the differences between the samples in the first and fourth quartiles. Two-way analysis of variance with Tukey’s test correction was used for multiple comparisons. A confidence interval of 95% was used for all the tests.

### 2.3 Immunohistochemistry

Immunohistochemistry was performed on a robotic platform (Ventana discover Ultra Staining Module, Ventana Co., Tucson, AZ, USA) by the Biorepository and Tissue Research Facility at the University of Virginia. Two tissue sections (4 µm) per sample were deparaffinized using EZ Prep solution (Ventana). One section was stained with hematoxylin (Fisher Scientific, Cat # 22-050-112) and eosin (Sigma, Cat # HT110132-1L). For triple-stained tissue sections, a heat-induced antigen retrieval protocol set for 64 min was carried out using a TRIS– ethylenediaminetetracetic acid (EDTA)–boric acid pH 8.4 buffer (Cell Conditioner 1). Sequential triple staining was first started with blocking of nonspecific binding of the antibody by Casein buffer (Ventana) for 12 minutes and endogenous peroxidases were blocked with peroxidase inhibitor (CM1) for 8 min before incubating the section with an anti-plectin antibody (Abcam, Cat # a 32528, RRID: AB_777339) at 1:3, 200 dilution for 60 minutes, with OmniMap anti-rabbit HRP detection and DISCOVERY ChromoMap DAB kit, the slide was then incubated with anti-CD8 antibody (Agilent, Cat #M7103, RRID: AB_207553) at 1:75 dilution for 60 minutes with DISCOVERY DISCOVERY anti-mouse HQ HRP detection system and DISCOVERY Purple kit, then with anti-CD68 antibody (Ventana Medical Systems Cat# 790-2931, RRID: AB_2335972) at 1:10 dilution for 60 minutes with OmniMap anti-Mouse HRP detection kit and DISCOVERY teal kit. Serial sections of each tumor were taken and stained for hemotoxylin and eosin. All slides were dehydrated, cleared, and mounted, and scanned using the Aperio ScanScope (Leica Biosystems). To analyze, 15 total patient tissues were annotated on QuPath (v05.1) with the guidance of a licensed clinical pathologist (Stelow). 10–20 tumor regions per tumor sample were obtained for a total of 219 individual tumor regions. Tumors were not selected if they were directly adjacent to areas of necrosis or regions of tissue that were folded or torn. Patient tumor annotations were then imported into Python for further analysis. For each tumor annotation, the mean deconvoluted DAB stain intensity was calculated as the mean value across all pixels within the annotated tumor region. This information was used to score DAB intensity on a scale of 1 to 3. Additionally, the number of CD8^+^ and CD68^+^ cells within 100μm of each tumor region were calculated algorithmically using software designed by Simbiosys, Inc. (Chicago, IL) by first identifying the minimum distance of all of CD8^+^ and CD68^+^ cells to each tumor region, then counting the number of CD8^+^ and CD68^+^ cells within a radial distance of 100μm of the exterior of each tumor annotation boundary. Statistical analysis was performed using GraphPad Prism software (version 10, GraphPad Software, Inc., San Diego, CA). Significance was determined using the Kruskal-Wallis test followed by Dunn’s multiple comparisons.

### 2.4 Cell surface biotinylation

Cell surface plectin levels on murine KPC915 and KPC 4662 were determined by isolating cell surface proteins using the Pierce Cell Surface Biotinylation and Isolation Protein Kit (Thermo Fisher Scientific) per the manufacturer’s protocol. Eluate protein concentrations were standardized using the Pierce BCA Protein Assay Kit (Thermo Fisher Scientific) and were separated using precast 4–20% tris glycine eXtended polyacrylamide gels (Bio-Rad, Hercules, CA, USA) and transferred to PVDF membranes using the iBlot3 Transfer system (Thermo Fisher Scientific) Membranes were blocked with Odyssey Blocking Buffer (LI-COR, Lincoln, NE, USA) then probed with the following antibodies: anti-plectin (ProteinTech, 29170-1-AP) anti- alpha 1 sodium-potassium ATPase (Cell Signaling Technology, #3010) and anti-GAPDH (Cell Signaling Technology, #2118). Membranes were imaged on an LI-COR Odyssey Infrared Imager using the anti-rabbit IgG IRDye 800 conjugated secondary antibody (LI-COR, #926-32213).

### 2.5 scRNA-seq data acquisition and pathway analysis

Single-cell RNA sequencing data were obtained from a previously processed PDAC dataset (https://zenodo.org/records/14199536) (31) and analyzed using the Seurat package (v5.4.0). Cell populations were subset from the full atlas based on pre-existing cluster annotations into two compartments: malignant ductal cells (DUCTAL, CYCLING DUCTAL) and immune cells (TNK, CYCLING TNK, MYELOID, CYCLING MYELOID, B CELLS, PLASMA, and MAST). To enable tractable analysis of the malignant compartment, 30, 000 ductal cells were randomly subsampled prior to pre-processing. All cell subsets underwent standard preprocessing including log-normalization, identification of the top 2, 000 highly variable features, scaling, and principal component analysis. Dimensionality was assessed via elbow plot and cumulative variance, and uniform manifold approximation and projection (UMAP) was computed using the top 30 principal components.

Patient-level PLEC expression was quantified by computing mean log-normalized PLEC expression across all cell types and in ductal cells per patient. PLEC quartile labels were mapped onto immune cells using patient identifiers to enable comparisons across quartiles. For formal differential gene expression analysis of the malignant ductal compartment, raw counts were aggregated per patient within each PLEC quartile using Seurat’s AggregateExpression function, with each patient representing an independent biological replicate. This pseudobulk approach was employed to avoid pseudoreplication inherent to cell-level differential expression testing of single-cell data (32). Genes with fewer than 10 counts in fewer than 2 samples were excluded prior to testing. Differential expression between quartiles was determined using DESeq2. Mitochondrial and ribosomal protein genes were excluded from the analysis. GSEA was performed against the Reactome pathway database using the gseaPathway function from the ReactomePA package (v1.54.0) with a minimum gene set size of 15, maximum of 500, and Benjamini-Hochberg adjusted p-value threshold of 0.05. Normalized enrichment scores (NES) were used to assess the direction and magnitude of pathway enrichment, with a positive NES indicating enrichment in the higher PLEC quartile relative to Q1. Differences in cell type proportions and module scores between PLEC^High^ and PLEC^Low^ patients were assessed using the Wilcoxon rank-sum test, with multiple testing corrections applied using the Benjamini-Hochberg method where applicable.

Immune cell type proportions were computed at the patient level by calculating the percentage of each immune cluster relative to the total immune cell count per patient, with zero counts explicitly assigned to patient-cluster combinations with no observed cells. Total T cell abundance was defined as the proportion of CD3^+^ cells within the TNK and Cycling TNK compartments relative to all immune cells per patient. The T cell lineage was assigned at the single-cell level using a multi-gene signature approach to mitigate the effects of CD4 transcript dropout inherent to single-cell RNA sequencing. CD8^+^ identity was scored using the mean expression of CD8A, CD8B, GZMB, GZMK, and NKG7, whereas CD4^+^ identity was scored using the mean expression of CD4, IL7R, TCF7, CCR7, FOXP3, and IL2RA, with cells assigned to the lineage with the highest mean signature score. T cell and myeloid subtypes were defined using curated literature-derived gene signatures (33–35). Module scores for T cell and myeloid functional states were computed using Seurat’s AddModuleScore function and averaged at the patient level for pseudobulk comparisons between the PLEC^High^ and PLEC^Low^ groups.

### 2.6 Subcutaneous mouse model

The following procedures were approved and conducted in accordance with the University of Virginia’s Institutional Animal Care and Use Committee (n=4) and Wuxi Biologics (n=2) for a total of (n=6). KPC915 cells previously described (36) and generously provided to us by the Kashatus Laboratory (University of Virginia) were maintained in KPC915 Medium (10% FBS/1% penicillin-streptomycin/DMEM) at 37 °C and 5% CO^2^ until injection. Cells were washed and resuspended in DPBS at 2 × 10^7^ cells/mL, then diluted in Matrigel membrane matrix (Fisher, CAT# CB-40234) to 1 × 10^7^ cells/mL The KPC915 tumor cell suspension was subcutaneously injected into one rear flank of female C57BL/6 mice (RRID: IMSR_JAX:000664) in a volume of 100 μL/mouse for a total of 1x10^6^ cells/mouse. Once tumors reached 100 mm^3^, mice were randomized into treatment groups and dosed intravenously in the tail vein with 3.0 mg/kg of αCSP mouse monoclonal antibody (clone 1D7) (16, 22) or IgG1κ isotype control (Bio X Cell Cat# BE0083, RRID: AB_1107784), on days 0, 3, and 7. Tumor growth was monitored and measured using calipers. The length (longest diameter) and width (shortest diameter) of the tumors were measured approximately twice weekly using calipers, and the tumor volume was calculated. The animals were continuously monitored for overall health, body weight, and tumor volume using the standard equation V = (L × W²)/2. Animals were euthanized if the tumor burden was in excess of 1000 mm³, ulcerated, or upon displaying clinical signs of severe illness, including weight loss >10%, inability to obtain food, extreme lethargy, failure to return to normal activity, or developing clinical signs necessitating euthanasia. The animals were euthanized using CO^2^ (30%-70% flow rate until fully anesthetized and confirmed by toe pinch) followed by cervical dislocation.

### 2.7 Orthotopic mouse model

All animal studies were approved by OHSU’s Institutional Animal Care and Use Committee. Female C57BL/6 mice (RRID: IMSR_JAX:000664) used in this study were purchased from the Charles River Laboratories. The mice were anesthetized using continuous isoflurane anesthesia. The abdominal areas were shaved 24 hours before surgery. Buprenorphine SR was administered at 0.01 mg/kg intraperitoneally 15 minutes before surgery for pain management. The shaved abdominal region of the animals was swabbed with a Betadine scrub before cleaning with 70% alcohol. 1.0-1.5 cm left abdominal flank incision was made, and the spleen and adherent pancreatic tissue were exteriorized. 4000 tumor cells were implanted into the pancreas in a mixture containing 50% DMEM and 50% Matrigel. PDAC murine KPC4662 (Vonderheide Laboratory) was derived from the primary tumors of LSL-Kras^G12D/+^;LSL-Trp53^R172H/+^; Pdx-1-Cre (KPC) C57BL/6 mice (37). 12 to 14 days after implantation Ultrasound was used to detect and measure the tumors 12–14 days after implantation. Tumors that reached 10–11 mm^2^ were randomized into different groups to initiate the treatment. Four doses of αCSP (clone ZB002) mAb or control mIgG2a (Bio X Cell, Cat# BE0085, RRID: AB_1107771) at 10 mg/kg were administered by IP injection every 72 h.

For the CD8α depletion experiments, tumors that reached 10–11 mm^2^ were randomized into different groups to initiate treatment. One mg/kg of anti-CD8α (Bio X Cell Cat# BE0117, RRID: AB_10950145) or rat-IgG2b_k_ (Bio X Cell Cat# BE0090, RRID: AB_1107780) was delivered by IP as the first dose, followed by 500 μg every 5 days for a total of three injections. Depletion of CD8^+^ T cells in the peripheral blood was verified by flow cytometry. 72 hours after the first anti-CD8α or rat-IgG2b_k_ injection, the mice were treated with four doses of 10 mg/kg αCSP (clone ZB002) or mIgG2a control every 72 h, beginning on day 4. The tumor size was monitored using ultrasonography. Fourteen days after the start of treatment, the animals were euthanized as described above, and the tumors were weighed. The measurements of tumor weight and size were unblinded.

### 2.8 Immunofluorescence

After harvesting, KPC4662 tumors were fixed in formalin overnight at room temperature. Tissues were washed with PBS, processed, and embedded in paraffin before sectioning. 10-μm-thick tumor sections were deparaffinized, and antigen retrieval was performed using citrate. Sections were incubated with blocking buffer (5% normal goat serum, 2.5% BSA, 1X PBS) at room temperature for 1 hour. Sections were stained overnight with purified anti-Plectin antibody clone E398P (ab32528) at 4 °C, followed by incubation with goat anti-rabbit IgG-A555 for 1 hour at room temperature. Autofluorescence of the tissues was removed using the Vector Laboratories Vector TrueVIEW Autofluorescence Quenching Kit. After quenching, DAPI was applied for nuclear staining. Slides were mounted with VECTASHIELD medium and imaged with a ZEISS Apotome microscope within 48 hours.

### 2.9 Immune subset analysis via flow cytometry

Orthotopic tumors were harvested after cardiac perfusion and subjected to enzymatic digestion study endpoints. For subcutaneous tumors, tumor sections were shipped on ice to Dr. Lisa Coussens at Oregon Health and Science University for immune subset analysis using flow cytometry. Tumors were chopped into small pieces and incubated for 30 min with a digestion medium containing 1.0 mg/ml collagenase IV *(Gibco 17104019)*, 1.0 mg/ml trypsin inhibitor *(Gibco 17075029)*, and 5000U/ml DNaseI *(Roche 10104159001)*. The cell suspensions were washed and incubated with the antibodies listed in **Supplementary Table 2**. First, the cells were incubated with anti-mouse CD16/CD32 *(BDFcBlock 553141)* and live/dead dye *(Molecular Probe Live/Dead Fixable Blue Dead cell stain 50-112-1524)* for 20 min. The cells were then stained with an extracellular antibody cocktail for 30 min. For intracellular staining, cells were fixed and permeabilized using the eBioscience Foxp3/Transcription factor staining buffer set (00–5523–00), following the manufacturer’s instructions. Finally, cells were stained with intracellular antibodies for 30 min. The cells were analyzed using Cytek Spectral Aurora. A list of antibodies, dilutions used, and vendor/catalog information is available in the **Supplementary Material**.

### 2.10 Statistical analysis.

Statistical analyses were performed using R software (v4.5.2). Results from *in vivo* experiments were analyzed using GraphPad Prism software (version 10, GraphPad Software, Inc., San Diego, CA). Differences between two groups were compared using the Wilcoxon rank-sum test. Multiple medians were compared using the Kruskal-Wallis test, followed by Dunn’s multiple comparisons. For single-cell analyses, patient-level pseudobulk comparisons of immune cell type proportions, T cells, and myeloid module scores were performed using the Wilcoxon rank-sum test. Spearman’s correlation was used to assess the relationship between PLEC+ ductal cell abundance and the proportion of immune cells. Multiple testing corrections were applied using the Benjamini-Hochberg false discovery rate method. A two-tailed P value of <0.05 was considered statistically significant for all analyses. The exact value of n within the figures is indicated in the figure legends and/or methods.

## 3 Results

### 3.1 High plectin expression is associated with a pro-tumorigenic phenotype in PDAC

**Figure 1:**
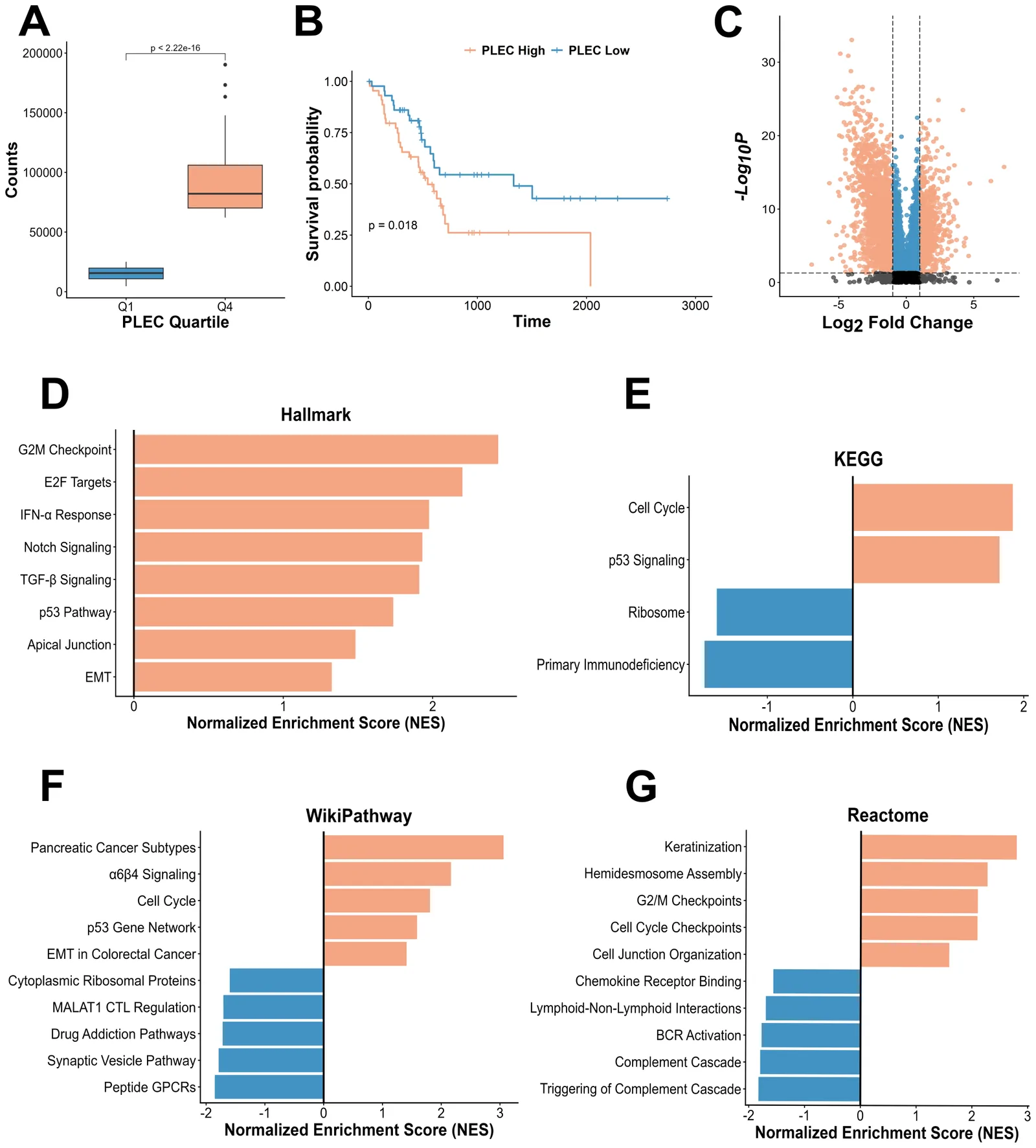
High plectin expression leads to worse survival and an aggressive phenotype in PDAC patients. **(A)** Normalized PLEC RNA expression counts from patients in the TCGA-PAAD dataset, categorized into quartiles. Significance assessed by Kruskal-Wallis test with Dunn’s *post-hoc* comparisons. **(B)** PLEC^High^ patients (pink; n=44) display significantly worse survival (p=0.018) when compared to PLEC^Low^ patients (blue; n=45). Groups were compared using the log-rank test. **(C)** Volcano plot of differentially expressed genes between PLEC^High^ and PLEC^Low^ samples. Differentially expressed genes were defined by |log_2_ fold change| ≥ 2 and adjusted p-value < 0.05. Points are colored as follows: pink, statistically significant, |log_2_FC| ≥ 2; blue, statistically significant (p < 0.05) |log_2_FC| < 2; grey, |log_2_FC| ≥ 2, p-value not significant; black, not significant. GSEA analysis from the Hallmark **(D)**, KEGG **(E)**, WikiPathway **(F)**, and Reactome **(G)**, gene sets from MSigDB identify pathways upregulated (pink) or downregulated (blue) in PLEC^High^ patients compared to PLEC^Low^ patients. PLEC^High^ patients display upregulation in pathways associated with PLEC function, as well as pathways associated with aggressive disease.

To understand the effects of plectin expression in PDAC, we analyzed 185 patient samples from the TCGA-PAAD dataset (38) and stratified the patients into quartiles based on their normalized PLEC RNA expression. The patient samples in the first quartile (PLEC^Low^) exhibited the lowest levels of PLEC expression, whereas those in the fourth quartile (PLEC^High^) exhibited the highest levels (**Figure 1A**; **Supplementary Figure 1A**). Subsequent Cox analysis revealed a significant decrease in survival within PLEC^High^ patients compared to PLEC^Low^ patients (p = 0.018; **Figure 1B**), underscoring the clinical relevance of plectin expression as a prognostic indicator in PDAC.

After stratifying the patient samples, DESeq2 analysis (24) was performed to identify differentially expressed genes (DEGs) between the two groups. A total of 1, 143 upregulated and 2, 823 downregulated DEGs were identified (**Figure 1C**; **Supplementary Table 1**). GSEA across multiple independent gene set databases retrieved from the Molecular Signatures Database (MSigDB) was evaluated: Hallmark (**Figure 1D**), KEGG (**Figure 1E**), WikiPathways (**Figure 1F**), Reactome (**Figure 1G**), and GO Biological Processes (**Supplementary Figure 1B**) (39). Consistent with the established role of plectin as a cytolinker, Reactome, and GO Biological Processes analyses further confirmed enrichment in structural pathways, including Keratinization, Hemidesmosome Assembly, and Cell Junction Organization (**Figure 1G**; **Supplementary Figure 1B**). Across all four databases, PLEC^High^ samples consistently displayed upregulation of pathways associated with cell cycle progression, including the G2M Checkpoint, E2F Targets, p53 Signaling and Cell Cycle Checkpoints (**Figures 1D–G**). Upregulation of invasive signaling pathways was also observed, including TGF-β Signaling, Notch Signaling, α6β4 Signaling, Pancreatic Cancer Subtypes, and EMT-associated pathways, across multiple databases, collectively reflecting an aggressive and invasive phenotype.

### 3.2 Plectin expression correlates with decreased anti-tumor immunity in patients

**Figure 2:**
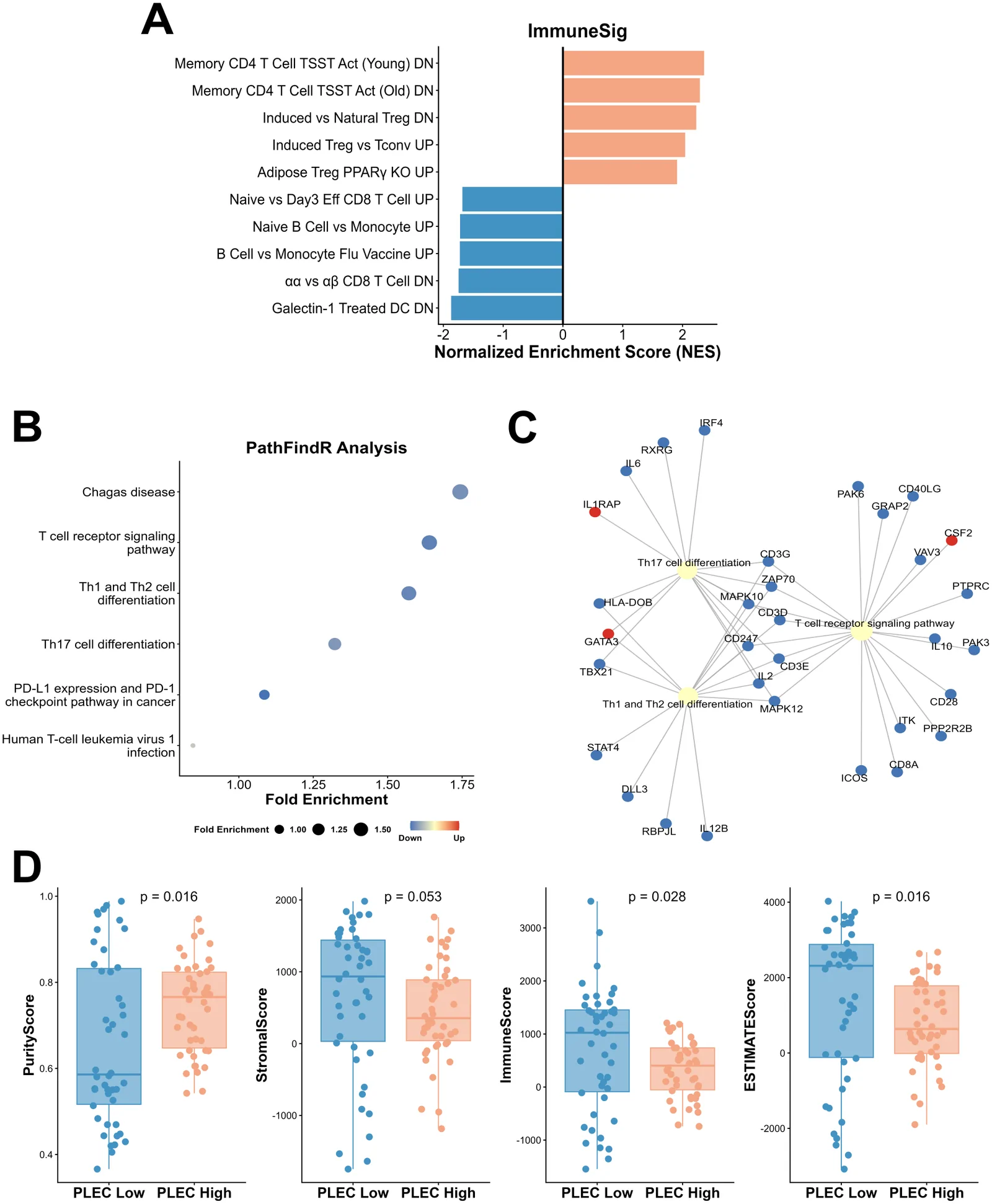
Plectin correlates with an immunosuppressive phenotype in PDAC. **(A)** GSEA analysis from the ImmuneSigDB gene set (MSigDB) suggests PLEC^High^ patients correspond with an immunosuppressive phenotype in cancer. **(B, C)** Pathway analysis using pathfindR after hierarchical clustering identifies suppression of immune-related pathways such as Th1 and Th2 differentiation, and T cell receptor signaling. Notable genes downregulated include CD3 genes, CD28, IL-6, and IL-12. **(D)** ESTIMATE analysis using tidyestimate identifies PLEC^High^ patients display a higher tumor purity score, and a significant decrease in immune and ESTIMATE scores. Wilcoxon rank-sum test.

Surprisingly, when evaluating gene sets, differential plectin expression correlated with immunoregulatory changes, where PLEC^High^ samples were associated with suppression of Chemokine Receptor Binding, Immunoregulatory Interactions Between Lymphoid and Non-Lymphoid Cells, and Complement Signaling when compared with PLEC^Low^ samples (**Figures 1F, G**). Analysis using the ImmuneSig gene set also identified DEGs associated with increased regulatory T cell (Treg) activity and decreased antitumor CD8^+^ T cell responses, indicating that PLEC^High^ patients may exhibit reduced antitumor immunity (**Figure 2A**). To further evaluate differential gene expression in PLEC^High^ versus PLEC^Low^ patients, we employed PathfindR, an active-subnetwork-oriented pathway enrichment analysis tool that incorporates protein–protein interaction networks in conjunction with gene expression data (40). Consistent with the GSEA results, PathfindR further illuminated the contribution of PLEC to cancer immunoregulation. PLEC^High^ samples showed downregulation of T cell signaling pathways, including the T cell receptor signaling pathway and Th1/Th2 cell differentiation (**Figures 2B, C**). We also observed downregulation of several inflammatory cytokines and their receptors, including IL-2, IL-6, STAT4, and CD3 complex proteins, such as CD3ϵ and CD3δ, which are classically responsible for antigen recognition and T cell activation (41) (**Figure 2C**). To investigate whether plectin expression directly influences the composition of the tumor immune microenvironment, we applied the ESTIMATE algorithm via the tidyestimate R package (42), which infers tumor purity and the degree of immune and stromal infiltration from bulk gene expression data. PLEC^High^ patients demonstrated significantly increased tumor purity (p = 0.016) and significantly reduced immune score (p = 0.028) and overall ESTIMATE score (p = 0.016) relative to PLEC^Low^ patients (**Figure 2D**), whereas stromal scores showed a decreasing trend that did not reach statistical significance in PLEC^High^ patients.

**Figure 3:**
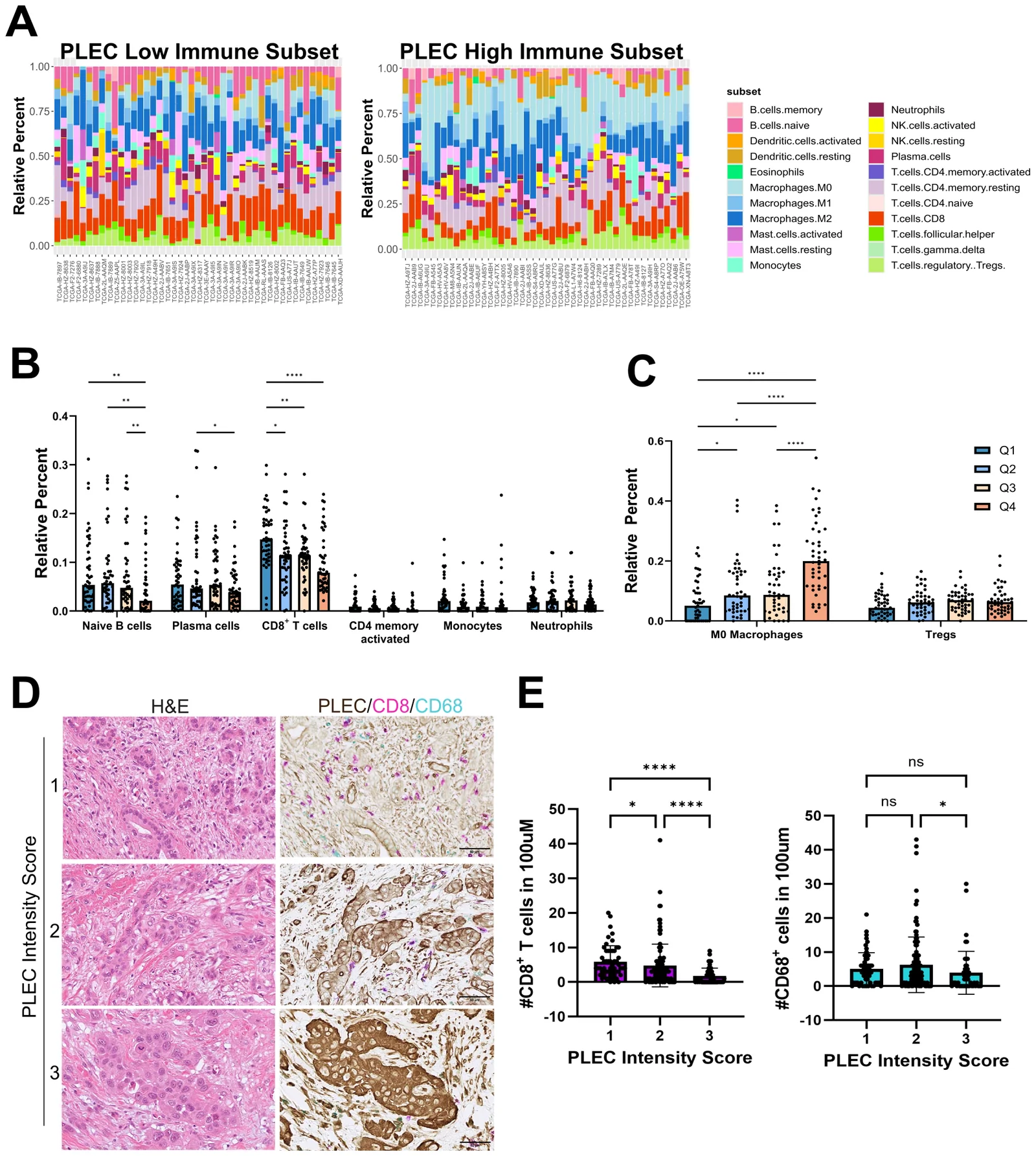
Immune deconvolution reveals that PLEC high patients have a significant decrease in CD8^+^ T cells. PLEC^Low^ and PLEC^High^ patients were evaluated using CIBERSORTx and the LM22 immune signature matrix. **(A)** Bar charts indicate relative percentages of the 22 immune subsets. **(B, C)** Relative percent scores of the 22 immune subsets for select immune cells reveal a significant decrease in naïve B cells, plasma cells, and CD8^+^ T cells, and an increase in M0 macrophages between quartiles. **(D)** IHC analysis of human PDAC specimens. H&E stain (left) and PLEC/CD8/CD68 triple stain (right) serial sections. Scale bar represents 50μm. **(E)** Quantification of the number of CD68+ and CD8+ cells within 100μm of selected tumor regions. Kruskall-Wallis test with Dunn’s multiple comparisons (*p<0.05, **p<0.01, ****p<0.0001).

Based on our bulk transcriptomic analysis indicating that PLEC^High^ patient samples display decreased anti-tumor immune signaling, we sought to more thoroughly evaluate the immune landscape of PLEC^High^ tumors. To accomplish this within patient tumors, we performed immune deconvolution using CIBERSORTx (30) and calculated the relative proportions of 22 immune cell subtypes using the LM22 matrix for each sample across all four quartiles (**Figure 3A**; **Supplementary Figure 2A**). The proportions of naïve B cells, plasma cells, and CD8^+^T cells were lower in PLEC^High^ samples, consistent with an immunosuppressed tumor microenvironment (**Figure 3B**). Moreover, the PLEC^High^ samples showed a significant increase in M0 macrophages and a slight increase in Tregs (**Figure 3C**).

To directly examine whether PLEC expression correlated with CD8 T cells or CD68-positive cells, 15 PDAC patient samples were stained for PLEC, CD8, and CD68. Tumor regions (n=10–20 per patient sample) were scored based on their PLEC expression on a scale of 1–3 based on the mean deconvolved DAB stain intensity by a board-certified pathologist (**Figure 3D**). The numbers of CD8^+^ and CD68^+^ cells within 100μm of each tumor region were calculated algorithmically by first finding the minimum distance of all CD8^+^ and CD68^+^ cells to each tumor region, then counting the number of CD8^+^ and CD68^+^ cells within a radial distance of 100μm of the exterior of each tumor annotation boundary. Although myeloid cells, specifically macrophages, make up a large proportion of the tumor microenvironment, we found that within a 100μm radius, tumors with higher PLEC expression show no major trend between groups (however, a significant reduction in CD68+ cells was seen between scores 2 and 3 (**Figure 3E**). Tumors with high PLEC expression contained significantly fewer CD8^+^ T cells within a 100μm radius compared to tumors that scored 1 or 2 (p<0.0001) (**Figure 3E**). This revealed that not only do PLEC high tumors contain fewer CD8^+^ T cells overall, but there were also fewer CD8^+^ T cells within direct vicinity of tumor regions. In contrast, PLEC low tumors showed a significant increase in the number of CD8^+^ T cells within the direct tumor vicinity.

Taken together, ESTIMATE scores, immune deconvolution analysis, and IHC analysis indicate that PLEC^High^ tumors correlate with an “immunologically cold” phenotype, while PLEC^Low^ tumors instead correlate with an “immunologically hot” phenotype (5, 6). However, the specific mechanisms by which plectin drives or is associated with this altered immune response remain unclear.

### 3.3 Single-cell analysis reveals plectin inhibits cytotoxic T cell infiltration

**Figure 4:**
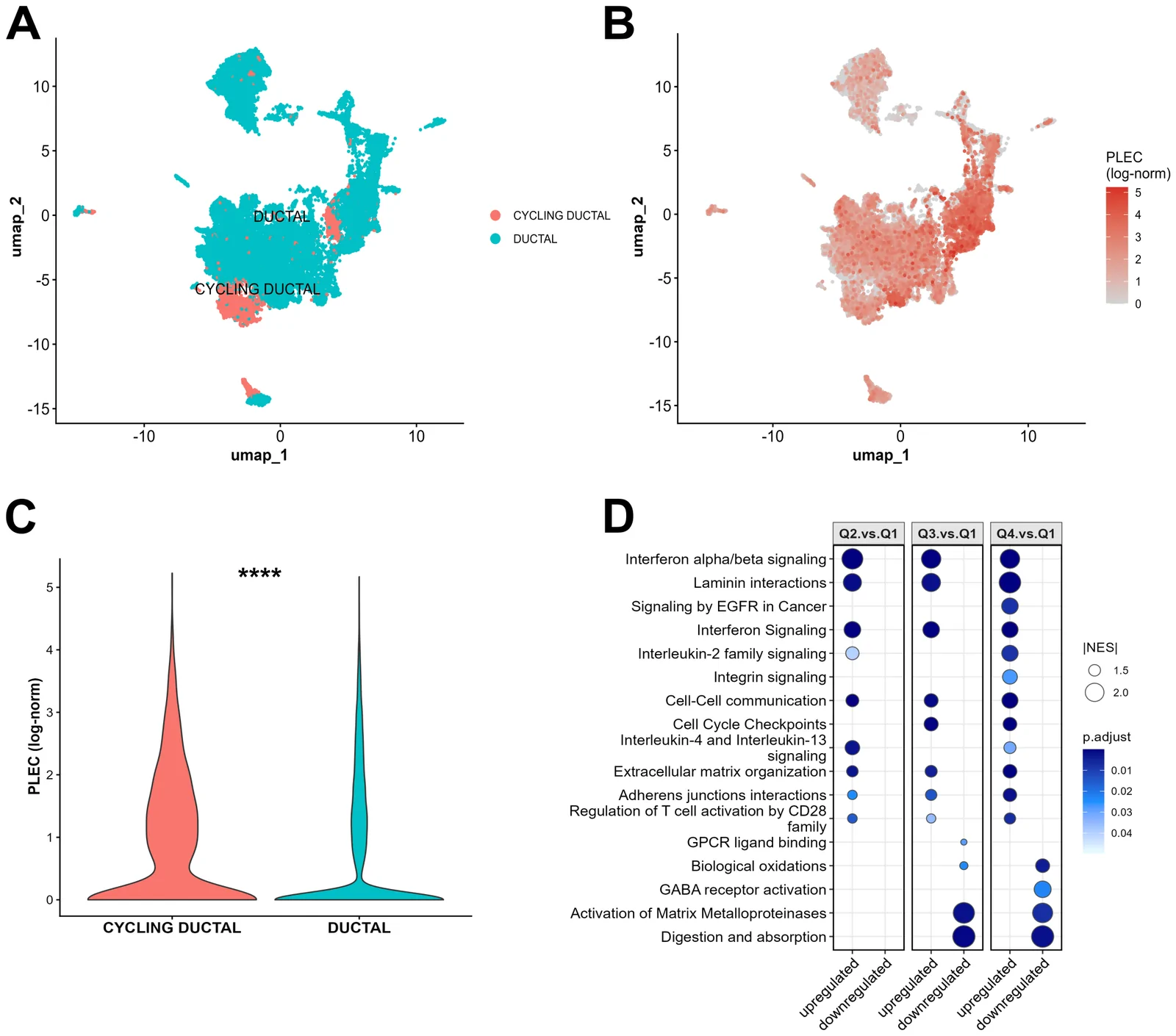
Single-cell analysis correlates plectin expression with highly proliferative and aggressive cells in PDAC subsets. Using a previously annotated dataset (31), ductal and cycling ductal cells were evaluated for PLEC RNA expression **(A, B)**. **(C)** PLEC expression was significantly upregulated in cycling ductal cells compared to ductal cells, suggesting high PLEC expression correlates with a more aggressive phenotype. Wilcoxon rank-sum test. ****p<0.0001. **(D)** GSEA analysis was performed against the Reactome database (MSigDB) to identify differentially enriched gene set pathways. Pseudobulked single-cell RNA-seq counts were quartiled by PLEC expression. Dot size reflects normalized enrichment score (NES), and color corresponds to BH-adjusted p-value. Across all quartiles, gene sets corresponding to an aggressive tumor phenotype were observed, as well as pathways suggesting immune suppression.

To more directly evaluate the immunosuppressive nature of plectin in PDAC, we analyzed a previously compiled scRNA-seq dataset containing samples from 229 patients (31). Ductal and Cycling Ductal subsets were isolated and evaluated for PLEC expression (**Figures 4A, B**). PLEC expression was high in both subsets, with a significant increase in the Cycling Ductal group (**Figure 4C**), consistent with our previous findings of aberrantly high PLEC expression in highly proliferative neoplastic cells (**Figure 1**). Furthermore, when evaluating all subsets for PLEC expression, Cycling Ductal and Ductal subsets show the most overall PLEC expression when compared to other clusters, with lower but detectable expression across stromal populations such as fibroblasts and endothelial cell populations (**Supplementary Figure 3**). To evaluate differences in PLEC expression levels, Ductal and Cycling Ductal single cell samples were pseudobulked to the patient level and stratified into quartiles, similar to bulk-RNA sequencing by mean PLEC expression, and GSEA was performed to compare each of the upper three quartiles to the lowest quartile (PLEC^Q1^) samples. DEG’s in each quartile were subsequently evaluated using Reactome pathway gene sets.

As in our bulk analysis, high PLEC expression was correlated with upregulated genes related to previously known plectin functions, such as Laminin Interactions, Integrin Signaling, Cell-Junction Organization, and Cell-Cell Communication pathways (**Figure 4D**). High plectin levels were also found to correlate with pro-tumorigenic pathways, including Cell Cycle Progression and Extracellular Matrix Organization across all quartiles, with effects more pronounced in PLEC^Q4^ cells. Interestingly, both Interferon Signaling and Regulation of T cell activation were upregulated across all quartiles (**Figure 4D**). Notably, the concurrent upregulation of Interferon Signaling and Regulation of T cell activation pathways in plectin-high tumor cells indicated a paradoxical immune activation state, wherein tumor-intrinsic interferon signaling may drive the expression of immune checkpoint ligands such as PD-L1, ultimately dampening rather than promoting cytotoxic T cell function (43, 44).

**Figure 5:**
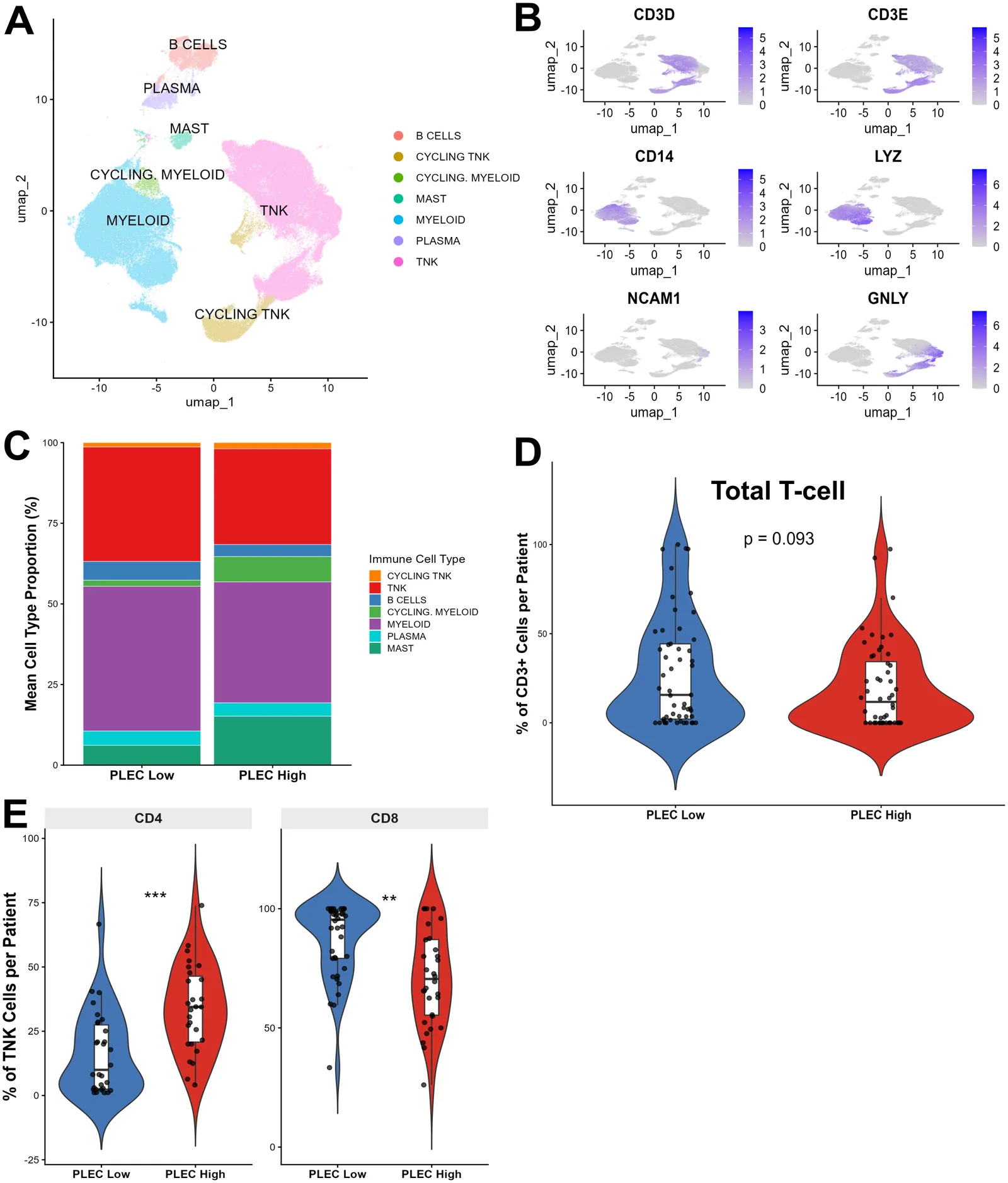
Immune subset analysis identifies plectin high patients displaying reduced CD8^+^ T cells in PDAC patients. **(A)** UMAP plot showcasing immune cell signatures within the immune compartment of the *Loveless et al.* dataset (31). **(B)** Expression of specific gene signatures for distinct immune cells were evaluated by feature plot within the immune subset. T cells were identified by CD3ϵ and CD3δ expression, myeloid cells by CD14 and LYZ, and NK cells by NCAM1 and GNLY markers. **(C)** Mean cell type proportion of each immune subset was evaluated between PLEC^Low^ and PLEC^High^ patients, revealing a decrease in TNK compartments and an increase in Cycling Myeloid and Mast cell compartments. **(D)** Patient-level pseudobulk quantification of total T cell abundance via RNA expression of CD3 in TNK and Cycling TNK clusters reveals a trending decrease in T cell population in PLEC^High^ patients when compared to PLEC^Low^ patients. **(E)** Within the TNK and Cycling TNK compartments, PLEC^High^ patients display an increase in the proportion of CD4^+^ T cells and a corresponding decrease in CD8^+^ T cells relative to PLEC^Low^ patients, indicating a shift in the cytotoxic to helper T cell balance. Wilcoxon rank-sum tests **p<0.01, ***p <0.001.

We further explored the relationship between plectin and tumor immune cell communication by analyzing the single-cell immune compartment. Annotated immune cells were normalized for compartmentalization via UMAP dimensionality reduction (**Figure 5A**), and the expression of canonical markers for distinct immune cell populations in the TNK and Myeloid compartments, including T cell markers (CD3ϵ, CD3δ), myelomonocytic markers (CD14, LYZ), and natural killer (NK) cell markers (NCAM1 and GNLY; **Figure 5B**) (33–35). Comparison of the mean immune cell proportions between lowest and highest PLEC quartiles (PLEC^Low^ and PLEC^High^, respectively) revealed a marked decrease in B cells and TNK Cells, along with a concurrent increase in Cycling Myeloid Cells and Mast Cell compartments in PLEC^High^ patients (**Figure 5C**). Given the observed shifts in broad immune compartments, we investigated the TNK and myeloid populations in greater detail. Stratification of TNK and cycling TNK cells by total T cell population and by CD4 and CD8 expression revealed a trending reduction in T cells (**Figure 5D**), a significant increase in CD4 ^+^ cells (p < 0.001), and a significant decrease in CD8^+^ cells (p < 0.01) within the PLEC^High^ cohort (**Figure 5E**), whereas no significant change was observed when stratifying myeloid cell subtypes (**Supplementary Figure 4A**). Finally, the assessment of T cell phenotypic markers across PLEC^High^ and PLEC^Low^ patients revealed a modest, non-significant increase in both exhausted T cells (T_ex_) and Tregs in PLEC^High^ patients (**Supplementary Figure 5**), further supporting the notion that elevated plectin expression promotes a T cell-suppressive tumor microenvironment characterized by reduced cytotoxic cell recruitment.

### 3.4 Targeting cell surface plectin results in increased CD8^+^ T cell infiltration *in vivo*

**Figure 6:**
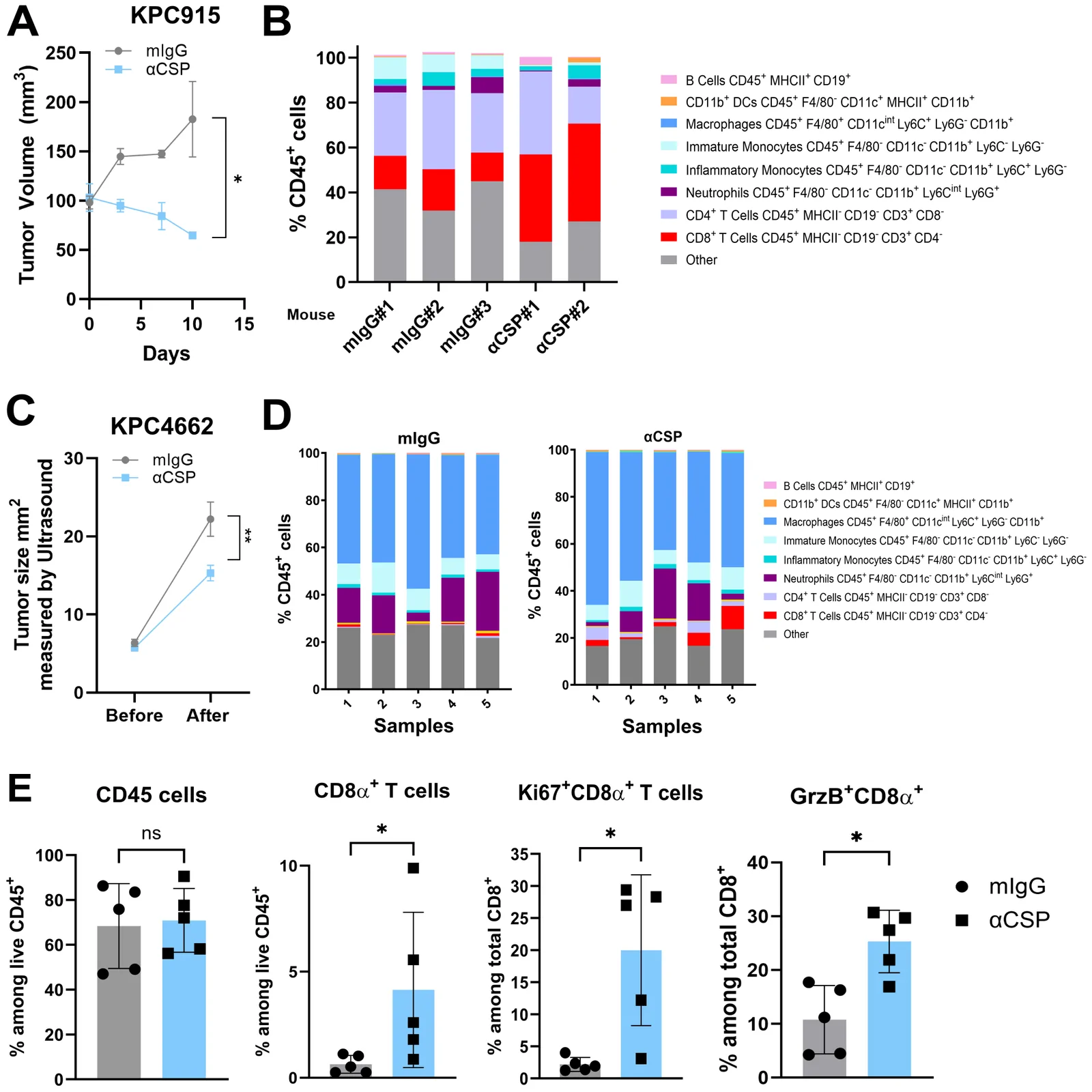
CSP inhibition causes tumor regression and CD8^+^ T cell infiltration. **(A)** Tumor growth analysis from mice injected s.c. with KPC915 and subsequently treated with mIgG isotype control (n=3) or 9.0 mg/kg/wk αCSP (n=2) shows a significant decrease in tumor volume over 10 days. **(B)** Tumors treated after one (αCSP#1) or two doses (αCSP#2) of αCSP or one dose of mIgG isotype control (mIgG#1, 2, 3) were digested to generate a single-cell suspension and stained with multiple antibodies via flow cytometry. Global immune infiltrates are shown as a percentage of CD45^+^ cells. Each bar represents an individual tumor. **(C)** Orthotopic syngeneic tumor cell transplantation of KPC4662 cells into the head of the pancreas and subsequent treatment of 10mg/kg mIgG (n=5) or 10mg/kg αCSP (n=5) shows a significant decrease in tumor size as measured by ultrasound. **(D, E)** After single-cell digestion, mice treated with αCSP show a significant increase in CD8^+^ T cells, including Ki67^+^ and Granzyme B^+^ (GrzB^+^) CD8^+^ T cells, showcasing proliferative anti-tumor activity after αCSP treatment. Wilcoxon rank-sum test. *p<0.05, **p<0.01.

To experimentally examine whether plectin contributes to the establishment of a T cell-excluded immune microenvironment and if an anti-tumorigenic immune response could be reinstated by therapeutically targeting plectin, we sought to leverage a previously evaluated neutralizing monoclonal antibody specific to Cell Surface Plectin (CSP) (16). To facilitate the accuracy of longitudinal tumor measurements, we used two different murine models of pancreatic cancer. Before implantation, two murine PDAC cell lines (KPC915 and KPC4662) were tested for CSP expression via cell surface biotinylation, with both cell lines having CSP expression (**Supplementary Figure 6A**). KPC915 cells were subcutaneously injected into the flank of C57/BL6 mice. Once tumors reached 100 mm^3^ in volume, mice were randomized into two treatment groups: 1) αCSP mAb at 9.0 mg/kg/wk, and 2) mIgG isotype control mAb at 9.0 mg/kg/wk. Mice received three doses in the first week, were monitored for tumor growth kinetics, and tumors were harvested for immune subset analysis. αCSP treatment resulted in >60% tumor regression on day 10, whereas treatment with IgG control antibody did not alter tumor growth (**Figures 6A**). To examine the changes in immune subsets after treatment, tumors were digested to generate single-cell suspensions and analyzed via flow cytometry using validated lineage-selective antibodies (**Supplementary Table 2**). Global immune infiltration was evaluated as the percentage of live CD45^+^ cells. As predicted from our *in silico* analysis, we found a significant increase in cytotoxic CD8^+^ T cells in tumors from αCSP-treated animals compared with IgG-treated controls (**Figure 6B**).

To investigate the action of plectin inhibition in a more clinically relevant model, we used a second murine-derived PDAC cell line, KPC4662, and implanted tumors orthotopically in the pancreas of C57BL/6 mice. Presence of CSP was validated using cell surface biotinylation and immunofluorescence, and tumor growth was monitored by ultrasound (**Supplementary Figures 6A, B**). Once tumors reached 10 mm^2,^the mice were randomized into two treatment groups: 1) αCSP mAb at 10 mg/kg and 2) IgG mAb isotype control at 10 mg/kg. Treatment occurred every three days, and 48 h after the last treatment on day 10, tumors were harvested and measured with calipers (**Figure 6C**). Treatment with αCSP at 10 mg/kg resulted in a significant decrease in tumor weight (p < 0.01) 12 days after the start of treatment. To examine the changes in immune subsets after treatment, the tumors were digested to generate single-cell suspensions (**Figure 6D**). As predicted from our CIBERSORTx analysis, scRNA-seq analysis, and subcutaneous model, αCSP treatment led to a trending increase in CD3^+^ T cells (p = 0.0556) and significantly increased the presence of CD4^+^ and CD8^+^ T cell populations (p < 0.05) (**Figure 6D**; **Supplementary Figure 6C**). Further analysis revealed that the CD4^+^ and CD8^+^ T cell populations in the PLEC-inhibited tumors were proliferating and actively cytotoxic, as evidenced by the expression of Ki-67 (p< 0.05) and Granzyme B (p < 0.05) (**Figure 6E**, **Supplementary Figure 6C**) (45).

### 3.5 Tumor regression from plectin inhibition is CD8^+^ T cell-dependent

**Figure 7:**
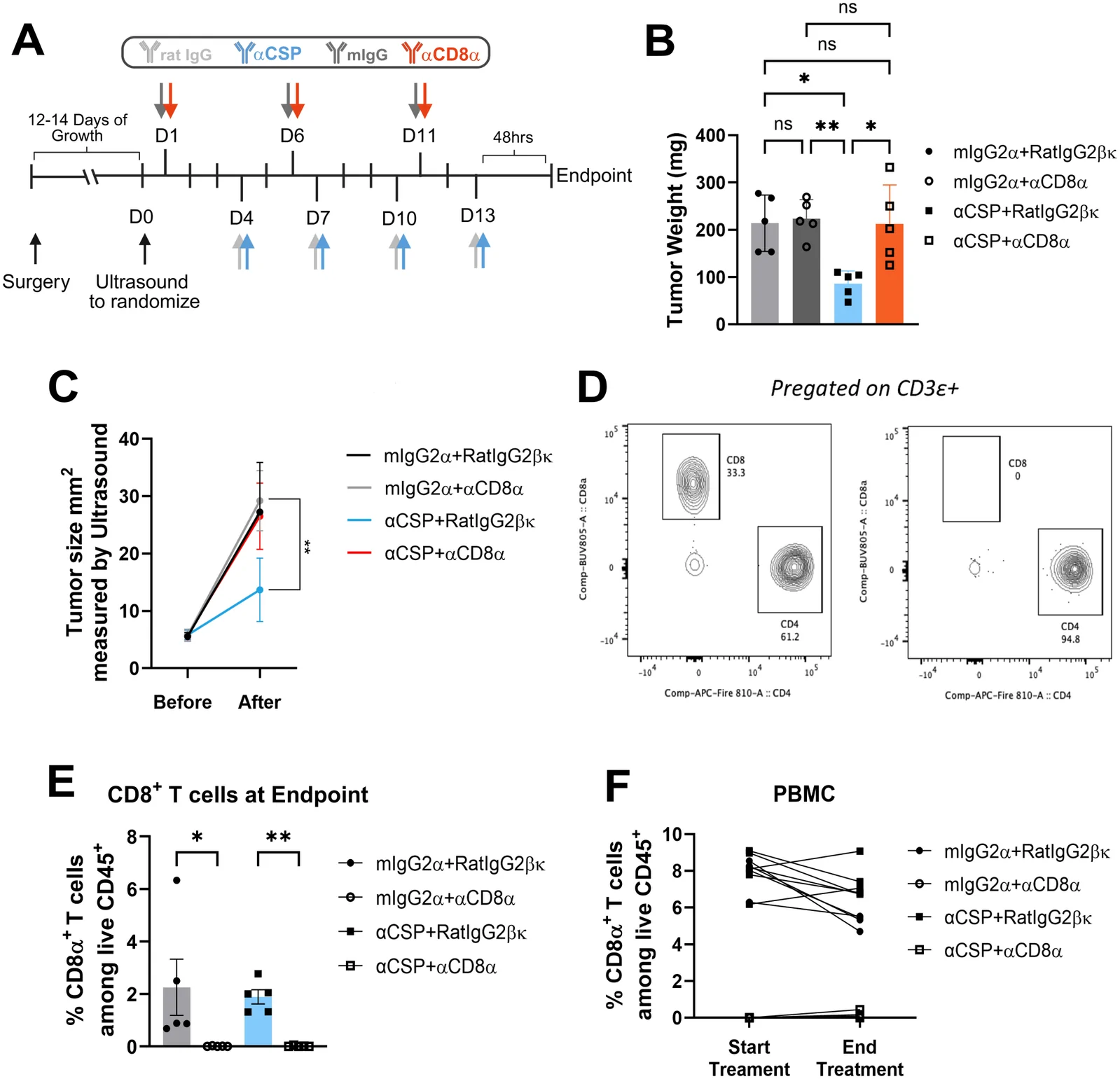
Anti-CSP-mediated tumor regression is CD8^+^ T cell-dependent. **(A)** Treatment schema. Mice were orthotopically implanted with murine KPC4662 cancer cells and subsequently treated with either rat IgG, mouse IgG, αCSP, or αCD8 on days specified (n=5 for all groups). **(B)** Tumor weight at study endpoint shows significant decrease in αCSP-treated mice and recovery of αCSP and αCD8-treated mice. **(C)** Tumor size measured by ultrasound confirms a significant decrease in tumor growth in αCSP-treated mice. **(D)** Flow cytometry analysis of CD8^+^ T cells, pre-gated on CD3ϵ^+^ T cells, before treatment and after treatment, shows a complete lack of CD8^+^ cells in mice treated with αCD8. **(E)** CD8^+^ T cell population as a percentage of CD45^+^ cells per treatment group. **(F)** Individual measurements of CD8^+^ T cells as a percentage among live CD45^+^ cells in PBMC before and after treatment. Error bars represent standard deviation. Kruskal-Wallis test. *p<0.05, **p <0.01.

Since targeting plectin with an αCSP mAb led to an increased presence of CD8^+^ T cells and subsequent tumor suppression, we sought to determine whether the antitumor effects were CD8^+^ T cell-dependent. Mice with orthotopically implanted KPC4662 tumors were randomized into four treatment groups: 1) mIgG + rat IgG, 2) mIgG + αCD8α, 3) rat IgG + αCSP, and 4) αCD8α + αCSP. Treatment was initiated once tumors reached 10 mm^2^, and CD8^+^ T cell depletion occurred two days before administering αCSP or mIgG isotype controls. The tumor weight and volume were measured 48 h after the final treatment (**Figure 7A**). As expected, depletion of T cells alone (mIgG + αCD8α) or both isotype control antibodies (mIgG + rat IgG) did not influence the tumor weight or volume. Rat IgG+αCSP treatment significantly reduced tumor weight compared to the mIgG + rat IgG or mIgG + αCD8α treatment groups (p < 0.05). Importantly, CD8^+^ T cell depletion before αCSP (αCD8α + αCSP) abrogated the antitumor effects of αCSP (**Figures 7B, C**). Cytometric analysis after pre-gating on CD3ϵ^+^ cells (**Figure 7D**) as well as measuring the fraction of CD8^+^ T cells among all CD45^+^ cells (**Figure 7E**) established that CD8^+^ T cells were, in fact, depleted by the treatment endpoint. Furthermore, the fraction of CD8^+^ T cells among all CD45^+^ peripheral blood mononuclear cells (PBMCs) before and after treatment confirmed the depletion efficiency (**Figure 7F**). These results demonstrate that the antitumor effects of αCSP treatment are dependent on CD8^+^ T cell cytotoxicity and affirm a critical role for CSP in impacting tumor immune evasion.

## 4 Discussion

Although personalized medicine has revolutionized cancer treatment and dramatically improved survival across many malignancies, the identification of clinically actionable targets that improve PDAC survival remains an unmet clinical need. This year, for the first time, the overall 5-year survival rate for all cancer patients reached 75%; however, for PDAC patients, this figure remains a disheartening 13%, a disparity that underscores the urgent need for new therapeutic strategies (1, 3, 4). This poor prognosis is largely driven by the paucity of effective systemic therapies, with surgery and chemotherapy being the primary treatment options for most patients. Compounding this challenge, PDAC has long been characterized as an immunologically “cold” tumor, rendering it largely refractory to immune checkpoint blockade therapies that have transformed the treatment landscape for other malignancies (4, 5). Identifying the mechanisms that underlie this immunosuppressive phenotype and therapeutic strategies capable of converting cold tumors to immunologically active ones represents a critical frontier in PDAC research. Here, we identified plectin as a novel immunomodulatory driver of the immunosuppressive tumor microenvironment in PDAC.

Our group previously employed a functional proteomic approach and revealed that plectin is aberrantly mislocalized to the cell surface of PDAC and other cancers, while remaining cytoplasmic in healthy tissues (7, 14, 15). Plectin expression has since been associated with worse overall survival across numerous malignancies, implicating it as an important mediator of tumorigenesis (10–13, 18, 19, 46). Building on these findings, we demonstrated that elevated plectin expression is directly associated with poor patient survival and an aggressive transcriptomic phenotype in the TCGA-PAAD dataset (**Figure 1**). Critically, PLEC^High^ patients displayed an “immuno-cold” gene expression signature (**Figure 2**), correlating with significantly decreased immune infiltration scores and a marked reduction in B and CD8^+^ T cells upon immune deconvolution and in patient samples via IHC (**Figure 3**).

To extend these findings to a higher-resolution platform, we leveraged a combined single-cell RNA-seq dataset previously annotated by *Loveless et al.* (31). PLEC expression was most strongly enriched in Cycling Ductal cells, a transcriptionally distinct ductal subset characterized by the high expression of STMN1 and HMGB2, markers associated with aggressive tumor biology (**Figures 4A–C**; **Supplementary Figure 3**). GSEA of ductal cell populations further confirmed an aggressive, pro-tumorigenic transcriptional program in PLEC^High^ ductal cells, consistent with our bulk transcriptomic analysis, including the upregulation of Laminin Interactions, Integrin Signaling, Cell Cycle Progression, and Extracellular Matrix Organization pathways (**Figures 1D–G**, **4D**). Notably, Interferon Signaling and Regulation of T cell activation were also upregulated in PLEC^High^ ductal cells across all quartiles, presenting an apparent paradox, given the immune-excluded phenotype observed in our single-cell immune analysis.

When examining plectin expression across all cell compartments, we observed notable expression within the fibroblast compartment (**Supplementary Figure 3**). This is expected given plectin’s canonical cytoskeletal function; however, it raises the question of whether plectin expression in fibroblasts gives rise to cell-surface plectin (CSP). To our knowledge, no study has specifically examined the presence of CSP on cancer-associated fibroblasts (CAFs). We have previously shown that CSP promotes exosome secretion in PDAC cells *in vitro*, which in turn increases binding of a CSP-targeting peptide (PTP) for both fibroblasts and endothelial cells (20). This suggests that, rather than arising from high plectin expression alone, CSP on fibroblasts may result from uptake of exosomes derived from CSP+ ductal cells. Future work should include analysis of CSP on CAFs and specifically examine whether anti-CSP therapies impair CAF function in addition to targeting tumor cells.

Interrogation of the immune compartment revealed that PLEC^High^ patients displayed a significant reduction in total T cell infiltration and concurrent expansion of myeloid populations (**Figure 5**). Further stratification by canonical immune markers demonstrated a significant increase in CD4^+^ T-helper cells (p < 0.001) and a significant decrease in CD8^+^ cytotoxic T cells (p < 0.01), corroborating the immunosuppressive phenotype observed in the bulk analyses (**Figure 5E**). T cells present in PLEC^High^ tumors displayed slightly elevated exhaustion module scores, marked by upregulation of checkpoint inhibitors, indicating that residual T cells were functionally impaired rather than immunologically active (**Supplementary Figure 5**).

Rather than reflecting productive antitumor immunity, the upregulation of tumor-intrinsic Interferon Signaling in PLEC-high ductal cells likely represents a paradoxical immune evasion mechanism. Interferon signaling plays pleiotropic roles in cancer, while acute interferon activation promotes anti-tumor immunity. Chronic or tumor-intrinsic interferon pathway activation can drive transcriptional resistance to immune checkpoint blockade through sustained upregulation of PD-L1, IDO1, and other immunosuppressive mediators (43, 44). Prolonged interferon exposure further promotes T cell exhaustion through the upregulation of inhibitory receptors (47, 48), consistent with the exhaustion phenotype observed in PLEC^High^ TNK cells.

Given that plectin has been found on the surface of cancer cells (CSP) and that CSP has been identified as a viable therapeutic target in various cancers (7, 14, 16) we hypothesized that plectin-mediated immune modulation is a direct consequence of CSP expression. Inhibition of CSP using a previously generated monoclonal antibody resulted in a significant tumor volume reduction in two independent murine PDAC models (**Figure 6**). Immune profiling of treated tumors revealed a significant increase in CD8^+^ T cells relative to IgG controls (**Figures 6B, D**), accompanied by robust expression of Ki-67 and Granzyme B, indicating that these cells were cytotoxically active (**Figure 6E**). This finding is particularly striking given that durable induction of T cell–mediated tumor killing remains one of the most significant barriers to effective PDAC treatment (6, 45).

To formally establish whether these antitumor effects were CD8^+^ T cell-dependent, we employed an *in vivo* depletion strategy in orthotopically implanted KPC4662 tumor-bearing mice (**Figures 7**). CD8^+^ T cell depletion prior to αCSP administration completely abrogated its anti-tumor effects, whereas depletion alone had no independent effect on tumor growth (**Figures 7B, C**). Depletion efficiency was confirmed both intratumorally and in PBMCs (**Figures 7D, F**), indicating that the loss of therapeutic efficacy was attributable to CD8^+^ T cell absence rather than incomplete depletion. Collectively, these data demonstrate that the antitumor activity of αCSP is CD8^+^ T cell-dependent, and CSP is a functionally significant mediator of immune evasion in PDAC, one whose pharmacologic inhibition is sufficient to restore cytotoxic T cell killing in a disease where T cell exclusion remains a defining barrier to effective immunotherapy.

Taken together, our findings establish plectin as a novel biomarker of immune exclusion and compelling therapeutic target in PDAC. Although our findings are the first to establish plectin as a negative immunomodulator in PDAC, independent corroborations have recently emerged. *Ge et al.* employed a machine learning-based approach to identify biomarkers distinguishing immunologically “cold” and “hot” PDAC tumors and independently identified plectin as a negative immune regulator (49), providing orthogonal validation of our central hypothesis. An important open question is whether the pro-tumorigenic and immunosuppressive effects observed here are driven by global plectin overexpression, aberrant CSP mislocalization, or both. Importantly, the specific isoform of CSP and the mechanistic underpinnings of how CSP operates on the cell surface remains to be discovered, making it difficult to currently differentiate the contributions of plectin and CSP. Disentangling these contributions will be critical for understanding the precise mechanisms by which plectin shapes the tumor immune microenvironment and for developing the most effective therapeutic approach. Further analysis of patient tumor samples for specific immune cell subtypes in PLEC^High^ samples will help unravel the precise mechanisms by which CSP represses CD8^+^ T-cell infiltration. Elucidating the precise mechanisms of CSP-mediated immune suppression will deepen our understanding of tumor microenvironment dynamics and may uncover additional therapeutic vulnerabilities in this devastating disease. In particular, direct examination of regulatory T cells and exhausted T cell populations via flow cytometry or spatial transcriptomics is critical to fully characterize CSP-mediated immune modulation and its therapeutic implications in PDAC. These results do suggest that anti-CSP treatment in combination with immune checkpoint inhibitors such as anti-PD1/PD-L1 inhibitors may improve outcomes for patients, however those experiments have yet to be performed.

In summary, this study identified plectin as a novel negative immunomodulator in PDAC, demonstrating that elevated plectin expression correlates with a T cell-suppressive tumor microenvironment characterized by reduced CD8^+^ cytotoxic T cell infiltration and broad suppression of pro-inflammatory immune signaling. Pharmacological targeting of cell surface plectin via αCSP monoclonal antibody was sufficient to restore CD8^+^ T cell-mediated anti-tumor immunity and significantly reduce tumor burden *in vivo*, in a manner that was strictly CD8^+^ T cell-dependent. These findings are of particular clinical relevance, given the notorious resistance of PDAC to existing immunotherapies and the continued lack of effective treatment options for patients with advanced disease. By establishing plectin as both a biomarker of immune exclusion and a functionally actionable therapeutic target, this study provides a compelling rationale for the continued development of anti-CSP therapies, either as standalone treatments or in combination with existing immune checkpoint inhibitors, with the potential to fundamentally shift the treatment landscape for one of the most lethal malignancies.

## Data Availability

Publicly available datasets were analyzed in this study. This data can be found here: Bulk RNA sequencing data are available from the Genomic Database Commons website (https://portal.gdc.cancer.gov/). Single-cell RNA-sequencing data are available at https://zenodo.org/records/14199536. All codes generated in this project to analyze the data are available at https://github.com/CodyWolf1/Plectin-represses-cytotoxic-T-cell-activity-in-pancreatic-cancer.git.
