# Supplementary material for: Plectin promotes an aggressive phenotype and represses cytotoxic T cell activity in pancreatic cancer
Source: Front Immunol. 2026 Aug 17;17:1894080. doi: 10.3389/fimmu.2026.1894080 (PMC13526953; doi:10.3389/fimmu.2026.1894080)
Supplement: Supplementary file 1 [file SupplementaryFile1.pdf]

Supplementary Material

Plectin promotes an aggressive phenotype and represses cytotoxic T cell activity in pancreatic cancer

Cody L. Wolf[1*], Roxanne K. Ruiz[1,2*], Sokchea Khou[3*], Robert Cornelison[4], Edward B. Stelow[4], Karl M. Kowalewski[5], Matthew J. Lazzara[1,5], Amanda Poissonnier[3], Lisa M. Coussens[3,6], Kimberly A. Kelly[1,2*†]

[1]Department of Biomedical Engineering, University of Virginia, Charlottesville, VA, USA.

[2] Department of Molecular Physiology and Biological Physics, University of Virginia, Charlottesville, VA, USA.

[3] Department of Cell, Developmental, & Cancer Biology, Oregon Health and Science University, Portland, OR, USA.

[4] Department of Experimental Pathology, University of Virginia, Charlottesville, VA, USA.

[5] Department of Chemical Engineering, University of Virginia, Charlottesville, VA, USA.

[6] Knight Cancer Institute, Oregon Health and Science University, Portland, OR, USA.

1 Supplementary Figures and Tables

1.1 Supplementary Figures

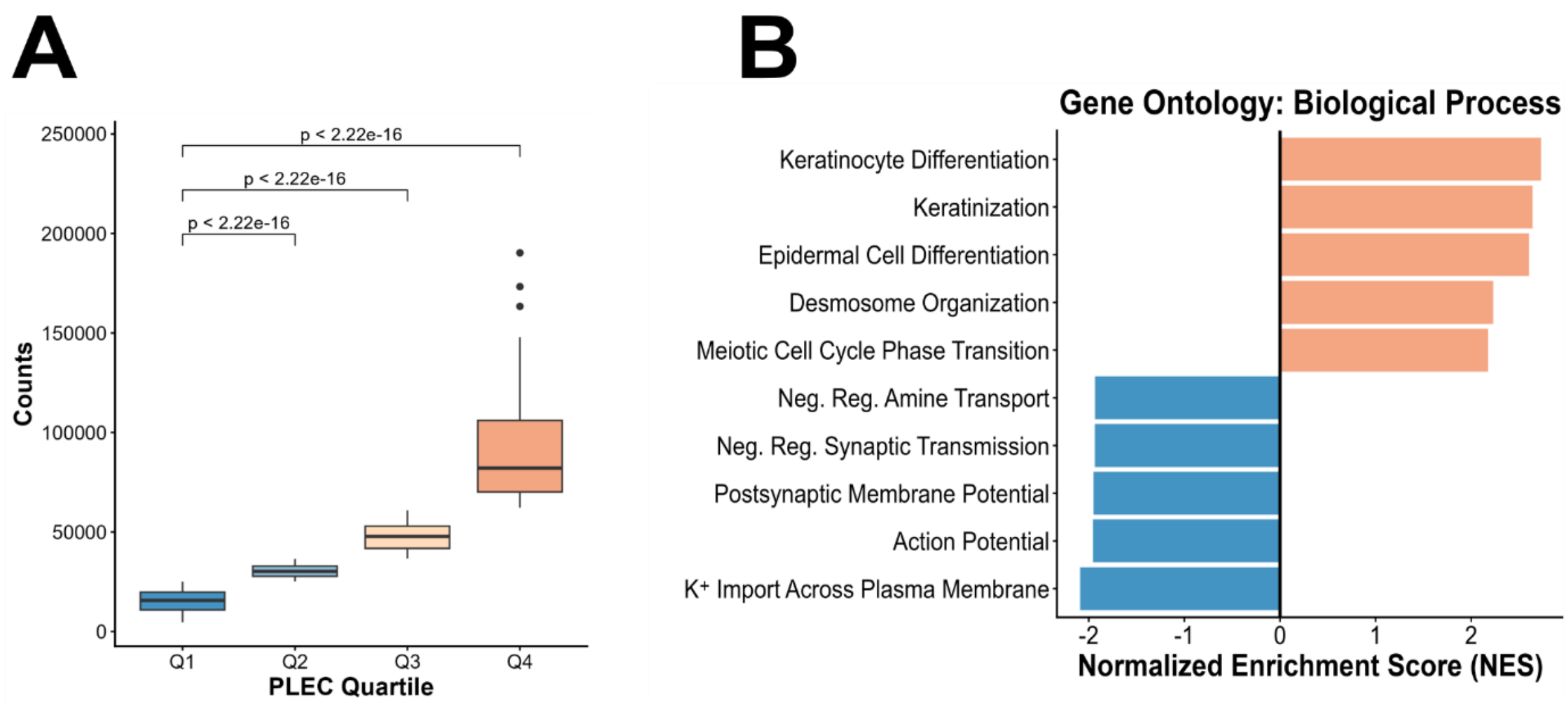

Supplemental Figure 1: Plectin expression corresponds with a more aggressive phenotype in PDAC. (A) Patients within the TCGA-PAAD dataset were categorized into quartiles based on normalized *PLEC* counts. Kruskal-Wallis test **(B)** GSEA of Gene Ontology: Biological Processes of upregulated (pink) and downregulated (blue) gene sets.

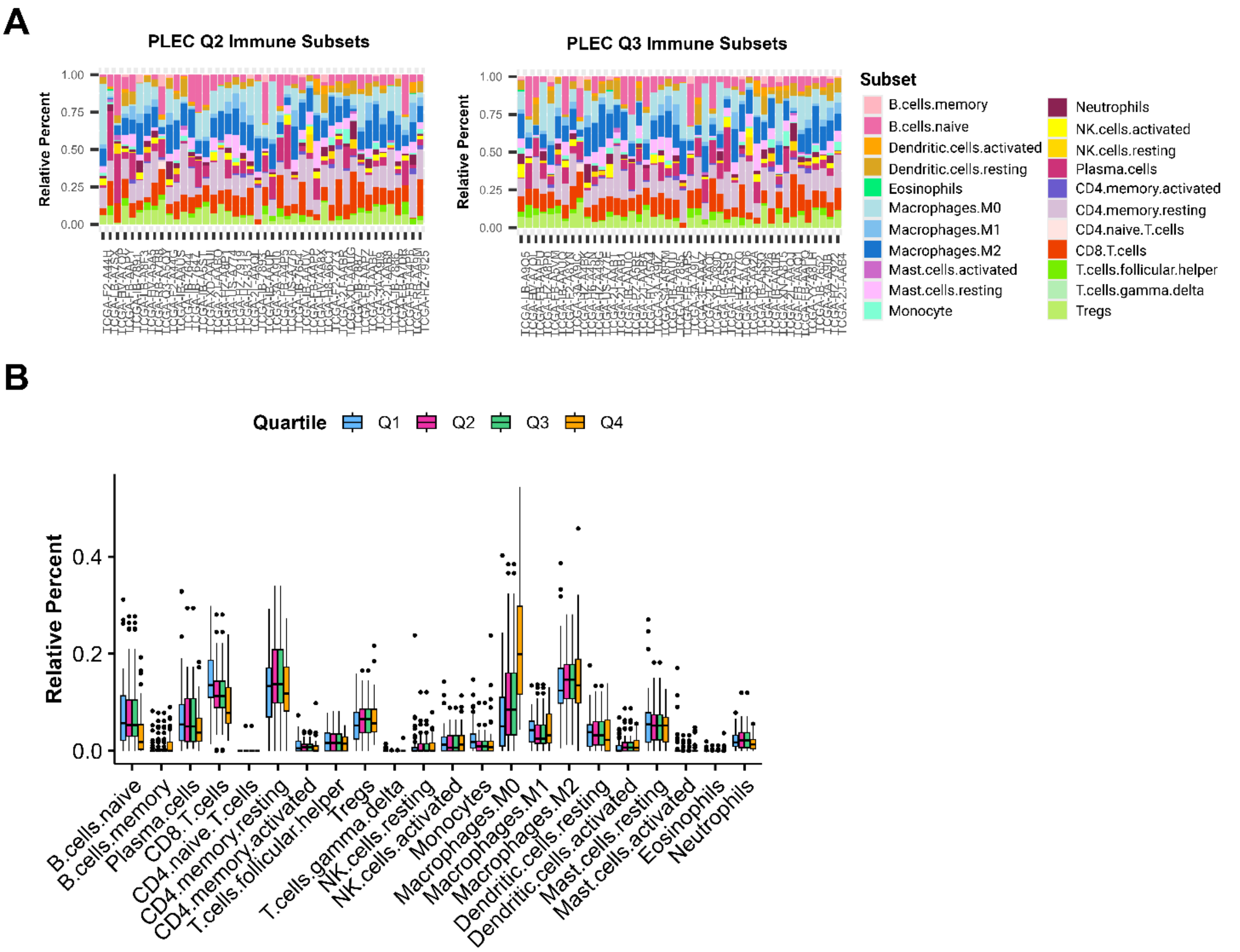

Supplemental Figure 2: Immune deconvolution of bulk PDAC data. PDAC patients from the second and third quartiles were run on the CIBERSORTx webserver using the LM22 immune signature matrix. (**A**) Bar charts indicate the relative percentages of the 22 immune subsets from Q2 and Q3 expressing samples. (**B**) Relative percent all 22 immune subsets from each quartile.

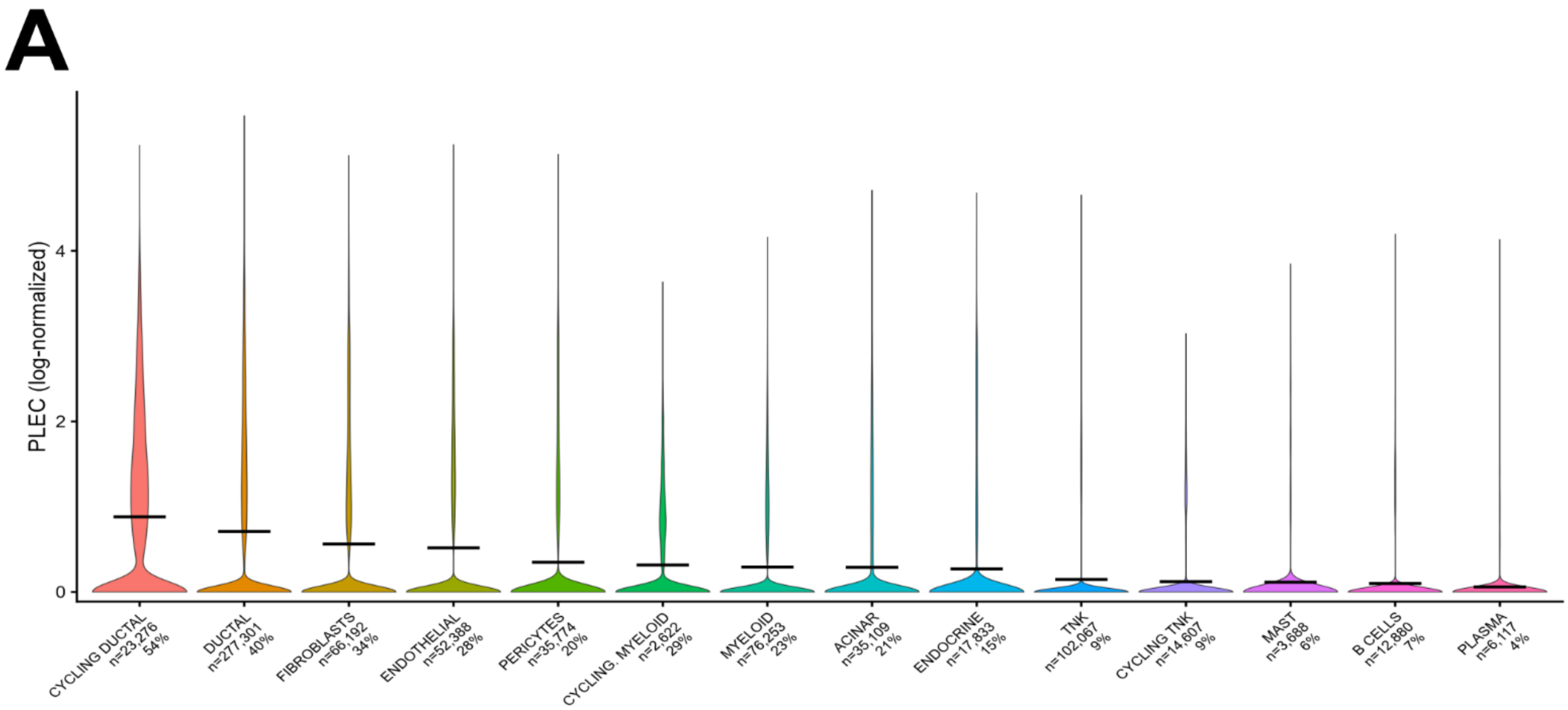

Supplemental Figure 3: Plectin expression across all scRNA-seq clusters. (A) Violin plots showing log-normalized PLEC expression in each annotated cluster within the *Loveless et. al.* dataset.Violin width is scaled to maximum width to aid visualization of distribution shape. Clusters are ordered by mean expression. Black crossbars denote mean, n= number of cells and % indicates percentage of cells with detectable PLEC expression in each cluster.

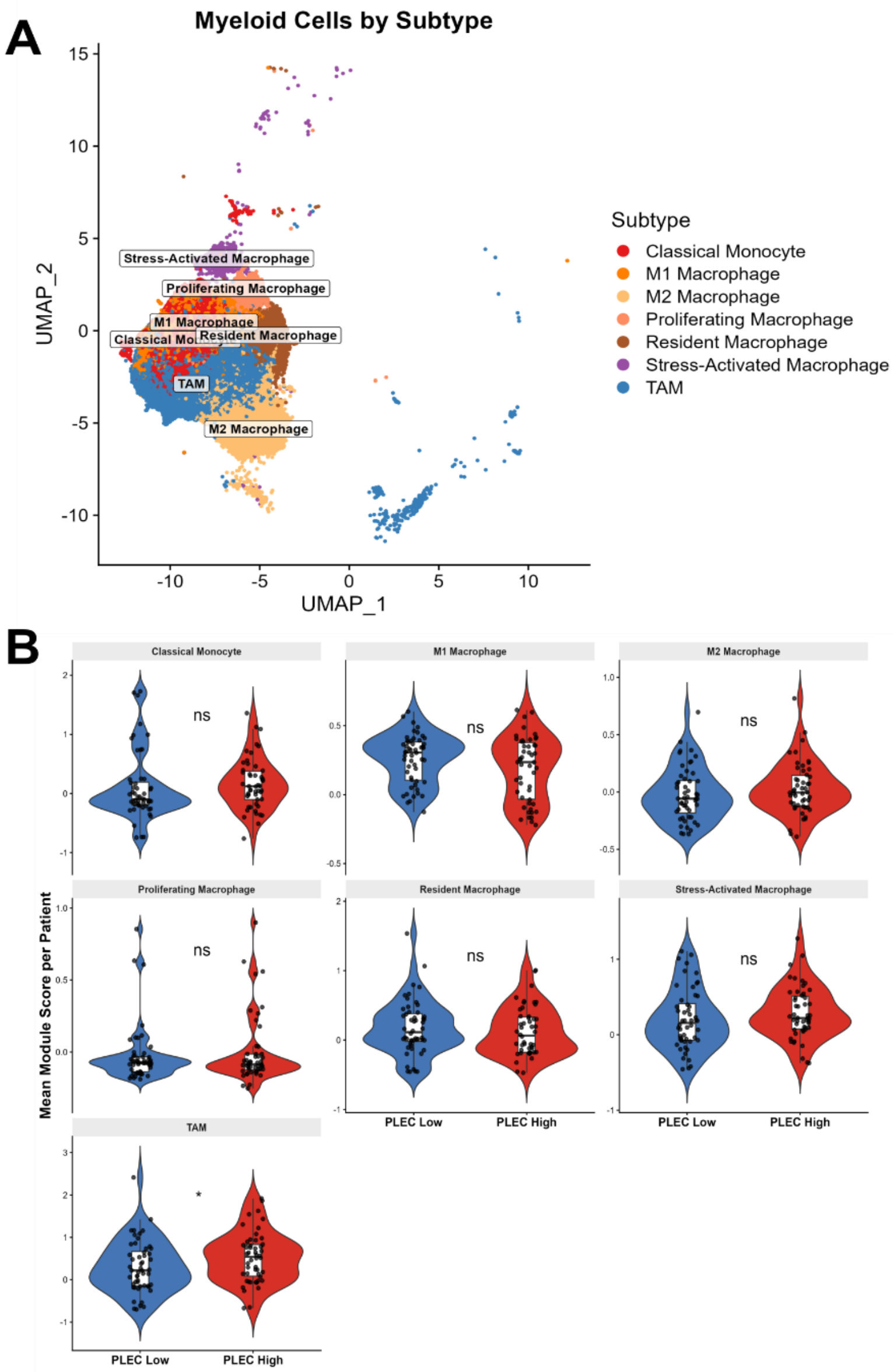

Supplemental Figure 4: Myeloid subtyping in PLEC^{High} vs PLEC^{Low} samples. (A) UMAP plot of myeloid subtypes based on genetic signatures. **(B)** Module scores of each myeloid subtype stratified by PLEC expression. Wilcoxon rank-sum test. ns = not significant.

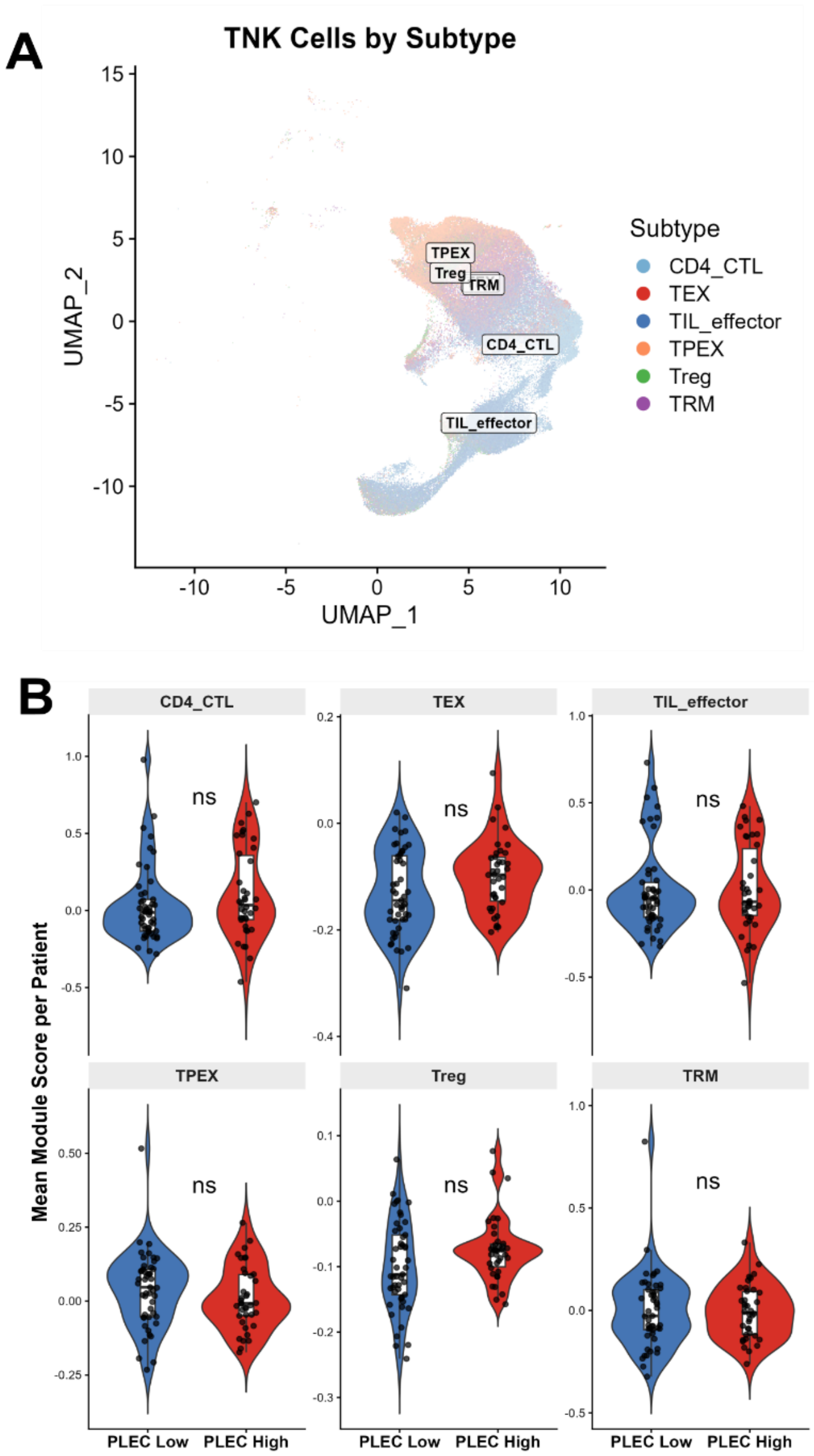

Supplemental Figure 5: T-cell subtyping in PLEC^{High} vs PLEC^{Low} samples. (A) UMAP of distinct T cell populations based on genetic signatures: terminally exhausted T cells (TEX), progenitor exhausted T cells (TPEX), effector TILs (TIL effector), cytotoxic CD4^{+} T cells (CD4 CTL), regulatory T cells (Treg), and tissue-resident memory T cells (TRM)**. (B)** Module scores of each T cell subtype in PLEC^{Low} and PLEC^{High} patients. Wilcoxon rank-sum test. ns = not significant.

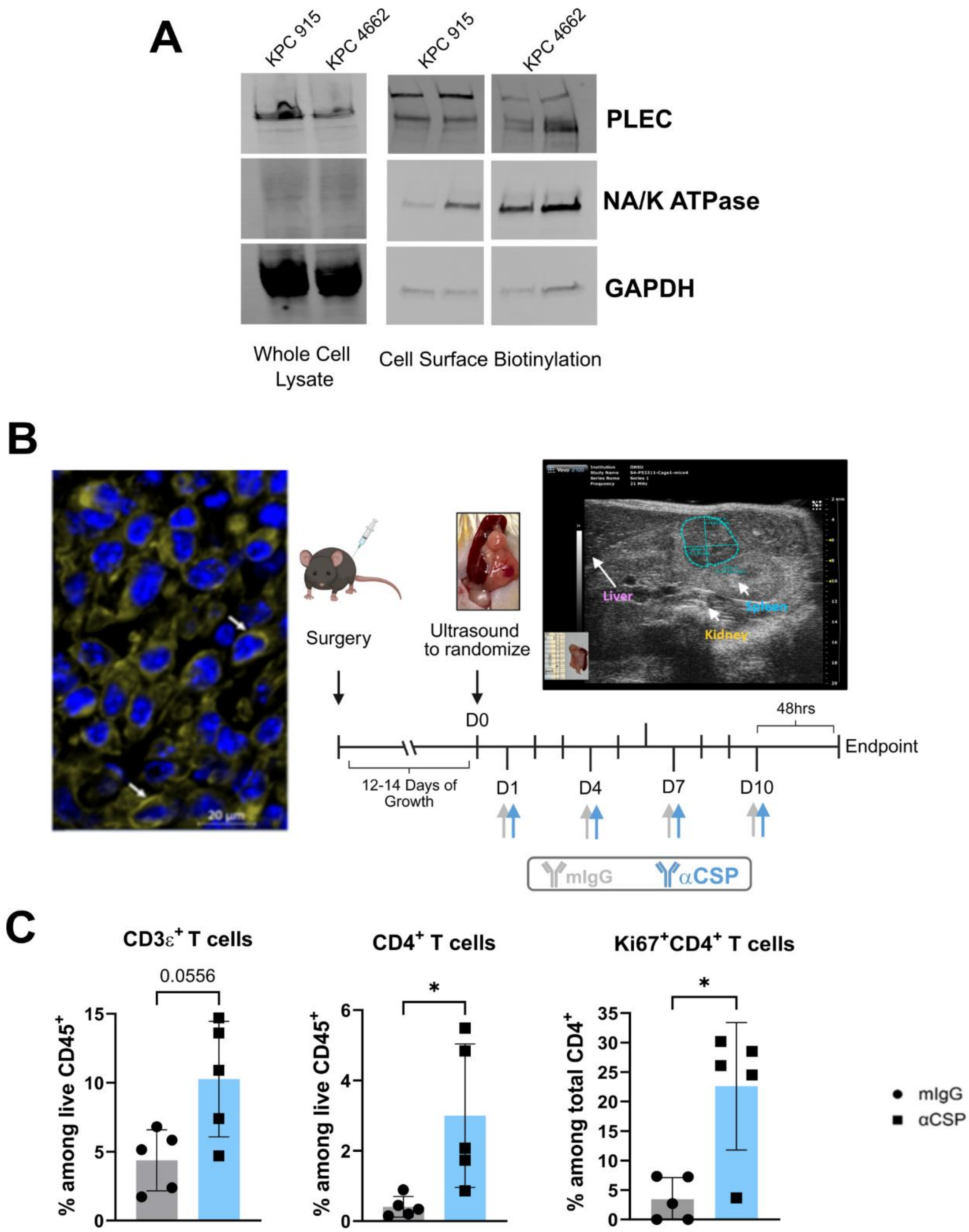

Supplemental Figure 6: Orthotopic PDAC model shows increased cytotoxic T cell infiltration post αCSP treatment. (A) Cell surface biotinylation of KPC915 and KPC4662 shows positive CSP-expression. NA/K ATPase and GAPDH were used as membrane and internal controls, respectively. **(B)** Treatment schema and ultrasound image showing tumor dimensions at beginning of treatment. **(C)** $CD3^{+}$, $CD4^{+}$ and $Ki67^{+}$ $CD4^{+}$ cells after treatment of either mIgG or αCSP. Wilcoxon rank-sum test. $^{*}p<0.05$.

1.2 Supplementary Tables

Fluorchrome	Marker	Dilution	Company	Cat#	RRID
BUV395	CD45	1000	BD Bioscience	564279	RRID:AB_2651134
BUV661	CD19	200	BD Bioscience	612971	RRID:AB_2870243
BUV737	CD3	400	BD Bioscience	612803	RRID:AB_2870130
BUV805	CD8	400	BD Bioscience	612898	RRID:AB_2870186
Super Bright 436	Siglec-F	200	eBioscience	62-1702-82	RRID:AB_2664259
eFluor 450	MHCII	1000	Thermofisher Scientific	48-5321-82	RRID:AB_1272204
BV480	*Ki67*	200	BD Bioscience	566109	RRID:AB_2739511
eFluor 506	*FoxP3*	100	Thermofisher Scientific	69-5773-82	RRID:AB_2637367
BV570	Ly6C	400	Biolegend	128029	RRID:AB_10896061
BV605	CD11c	400	Biolegend	117334	RRID:AB_2562415
BV650	NK1.1	50	Biolegend	108735	RRID:AB_11147949
BV750	CD103	200	BD Bioscience	747478	RRID:AB_2872154
Alexa Fluor 532	CD11b	400	Thermofisher Scientific	58-0112-82	RRID:AB_2811905
PE-Cy5.5	*GrazB*	100	Invitrogen	35889882	RRID:AB_2848329
Spark YG593	CD44	200	Biolegend	103078	RRID:AB_2892266
PE-CF594	PD-1	200	BD Bioscience	562523	RRID:AB_2737634
APC	CD206	100	Biolegend	141708	RRID:AB_10900231
Spark NIR 685	Ly6G	100	Biolegend	127666	RRID:AB_2876454
Alexa Fluor 700	CCR7	100	Thermofisher Scientific	56-1971-82	RRID:AB_657687
APC-Fire 750	F4/80	100	Biolegend	123152	RRID:AB_2616725
APC/Fire™ 810	CD4	200	Biolegend	100480	RRID:AB_2860583

Supplemental Table 2: Antibodies used for multi-color flow cytometry.
